# HFIP as a versatile solvent in resorcin[*n*]arene synthesis

**DOI:** 10.3762/bjoc.20.211

**Published:** 2024-10-02

**Authors:** Hormoz Khosravi, Valeria Stevens, Raúl Hernández Sánchez

**Affiliations:** 1 Department of Chemistry, Rice University, 6100 Main St., Houston, Texas 77005, USA; 2 Rice Advanced Materials Institute, Houston, Texas 77005, USA

**Keywords:** cavitand, cyclization, HFIP, hydroxyalkylation, resorcinarenes

## Abstract

Herein, we present 1,1,1,3,3,3-hexafluoroisopropanol (HFIP) as an efficient solvent for synthesizing resorcin[*n*]arenes in the presence of catalytic amounts of HCl at ambient temperature and within minutes. Remarkably, resorcinols with electron-withdrawing groups and halogens, which are reported in the literature as the most challenging precursors in this cyclization, are tolerated. This method leads to a variety of 2-substituted resorcin[*n*]arenes in a single synthetic step with isolated yields up to 98%.

## Introduction

**Scheme 1:**
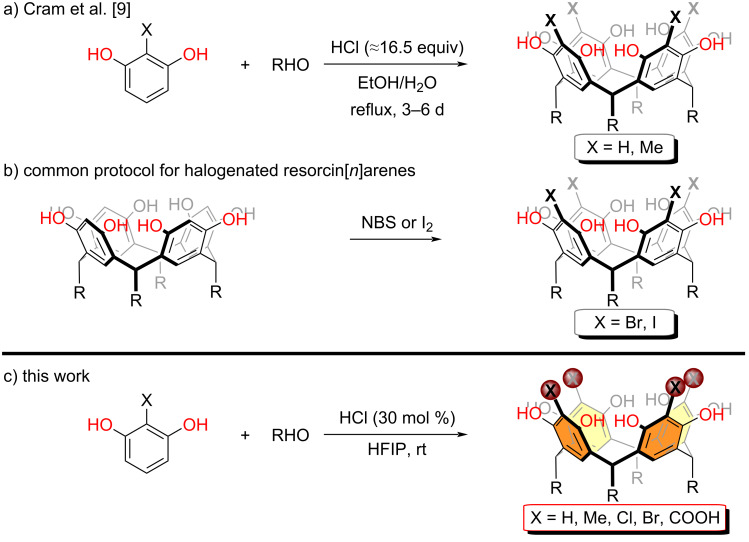
Resorcin[*n*]arene synthesis.

The acid-catalyzed aldehyde-resorcinol condensation has been studied for more than a century [1–3]. Decades of research culminated in the landmark paper by Niederl and Vogel [4], whose quantitative elementary analysis and molecular weight determinations led them to conclude that the most likely product of the aldehyde-resorcinol condensation was a four-fold species resulting from intermolecular dehydration, nowadays known as alkyl resorcin[4]arene. Forty years later in 1980, Höegberg noticed that short alkyl chain resorcin[*n*]arenes develop stereoisomers in the reaction mixture; however, since the condensation reaction is reversible, once the macrocycle adopts the bowl-shaped conformation it precipitates out of solution acting as a thermodynamic sink [5–6]. Shortly after, Cram et al. recognized the potential of resorcin[*n*]arenes as compounds large enough to encapsulate other simple molecules or ions and group them with other known macrocyclic arene compounds, e.g., spherands, cyclotriveratrylene, and calix[*n*]arenes, under a class termed cavitands [7–12]. The popularity of resorcin[*n*]arenes has grown by contributions from Diederich [13–17], Rebek [18–26], Gibb [27–33], Atwood [34–36], Szumna [37–39], Reinhoudt [40–41], Konishi [42–44], Tiefenbacher [45–52], Strongin [53–55] among others. The wide-ranging popularity of resorcin[*n*]arenes is rooted in the numerous applications these compounds have in supramolecular chemistry, e.g., catalysis and molecular recognition [12,46]. Despite extensive research, challenges remain in the acid-catalyzed resorcin[*n*]arene synthesis, for example: 1) reaction times for simple resorcin[*n*]arenes starting from aliphatic aldehydes and resorcinol generally require multiple days and up to a week (Scheme 1a) [9,26]; 2) use of 2-substituted electron-poor resorcinols with aldehydes larger than acetaldehyde produce intractable mixtures leading to no product isolation [9], in turn access to halogenated or deactivated electron-poor resorcin[*n*]arenes typically require an extra step as shown in Scheme 1b [8,56–59]; and 3) access to larger macrocycles with *n* > 4 is not a trivial task usually leading to reaction yields <10% [21,38,42,60]. Herein, we report 1,1,1,3,3,3-hexafluoroisopropanol (HFIP) as an efficient solvent to speed up reaction times and also capable of tolerating electron-deficient and halogenated 2-resorcinols in the synthesis of resorcin[4]arenes (Scheme 1c). Our work addresses the first two challenges highlighted before by providing several examples that will be useful to scientists in this research field.

Resorcin[*n*]arenes synthesis is generally high-yielding and straightforward. Their unique bowl-shape structures and self-assembly in solution have facilitated their widespread use as building blocks in nanomaterials [61–62]. Nevertheless, precisely due to their vast number of applications, functionalized resorcin[*n*]arenes are needed that meet the needs of the desired function. For example, halogen-containing resorcin[*n*]arenes are highly sought after as they engage in divergent synthesis [8,63–68]. However, 2-haloresorcinol does not cyclize under standard protocols (Scheme 1a) pushing the need for an additional halogenation step (Scheme 1b). To overcome this limitation, we analyzed the mechanism underlying the formation of resorcin[*n*]arenes. The first step of the cyclization reaction is a hydroxyalkylation involving various cationic intermediates [69]. Hence, we hypothesized that any factor enhancing the rate of the first step by stabilizing carbocations will likely enable new starting materials to be used in resorcin[*n*]arene synthesis. Since HFIP has recently gained popularity as a solvent capable of stabilizing carbocations in diverse type of reaction settings [70–72], we opted to utilize this solvent to overcome the constraints highlighted above (Scheme 1c).

## Results and Discussion

### Optimization

To test our hypothesis of using HFIP as a potent solvent in resorcin[*n*]arene synthesis, initial experiments were conducted using resorcinol and valeraldehyde (Table 1). To compare, Cram’s seminal report from 1989 [9] described the following conditions and results for resorcinol and valeraldehyde: 1:1 EtOH/H_2_O, 3 equiv HCl, rt, 6 days, 89%. We began by screening various solvents and combinations of solvents including HFIP. Those experiments revealed that the most favorable outcomes are achieved in the presence of HFIP in its pure form in the presence of HCl as the catalyst (Table 1, entries 1–4). The removal of HCl from the reaction conditions unveiled the crucial role of the catalyst in the process (Table 1, entry 5), which was expected; however, note that here we use the acid in catalytic amounts and not in excess as reported in the literature [73–75]. Variations in catalyst nature between a Brønsted and Lewis acid, and the acid’s p*K*_a_ (Table 1, entries 6–8) did not improve the yield compared to HCl (Table 1, entry 4). Last, further exploration of the conditions using HFIP/HCl revealed that the reaction progress achieves its maximum conversion early on at just 20 minutes (Figure S1 in Supporting Information File 1). Remarkably, these results demonstrate that reaction times can be decreased from 72–144 h [26] to approximately 1 hour.

**Table 1:** Optimization of resorcin[*n*]arene synthesis using HFIP.^a^

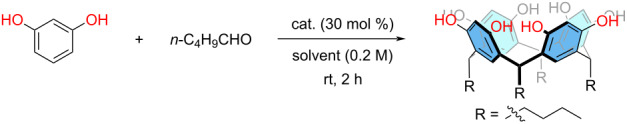			

Entry	Solvent	Cat.	Yield^b^ (%)

1	MeOH	HCl	0
2	HFIP/MeOH 1:1	HCl	35
3	HFIP/DCM 1:1	HCl	44
4	HFIP	HCl	92
5^c^	HFIP	–	0
6	HFIP	TFA	≈4
7	HFIP	TfOH	43
8	HFIP	Zn(OAc)_2_	0

^a^The reaction was performed with resorcinol (1.0 mmol), valeraldehyde (1.0 mmol), and catalyst (30 mol %) in solvent (5 mL) at room temperature for 2 h. ^b^Yields were determined by ^1^H NMR analysis of the unpurified reaction mixture using CH_2_Br_2_ as an internal standard versus the resorcin[4]arene’s methine resonance located at 4.17 ppm (triplet, *J* = 7.9 Hz, 1H). ^c^The reaction yields no product at room temperature or under reflux conditions. TfOH = triflic acid.

### Substrate scope

**Scheme 2:**
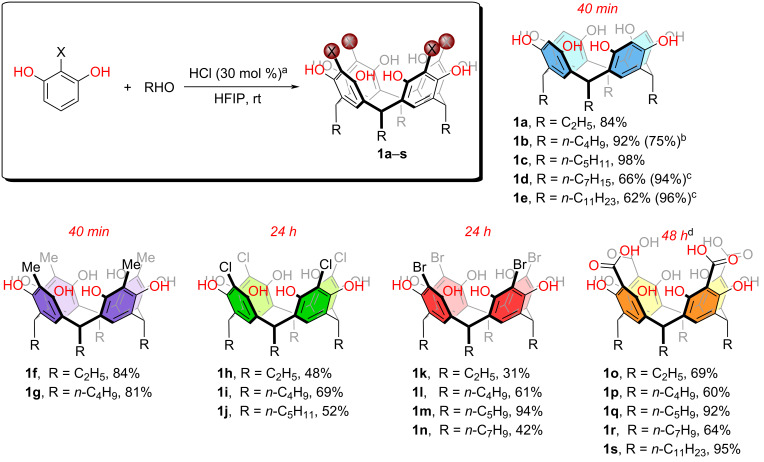
Scope of resorcin[*n*]arene synthesis using HFIP. ^a^All reactions were performed with resorcinol (1.0 mmol), aldehyde (1.0 mmol), and HCl (30 mol %) in HFIP (5 mL) at room temperature, and the yields of the reactions are given as isolated yields. ^b^Gram-scale reaction (1.10 g of resorcinol). ^c^Reaction time increased to 24 h. ^d^All reactions were performed at 50 °C.

To demonstrate the limitations and scope of the reaction, we first used resorcinol in the presence of various aldehydes featuring different alkyl chains, all of which exhibited high isolated yields (Scheme 2, compounds **1a**–**e**). Interestingly, literature reports commonly show lower yields as the aldehyde alkyl chain increases in length where commonly refluxing temperatures are required [9,74,76]. In contrast, the protocol reported herein provides 94–98% yield when employing longer chain-containing aldehydes (**1c**–**e**).

In addition to resorcinol, 2-methylresorcinol is commonly used in resorcin[*n*]arene synthesis as radical oxidation of the methyl unit in the Ar*CH_3_* fragments provides a benzyl synthon, which is used conveniently towards other applications, e.g., metal cluster synthesis, and halogen and hydrogen-bonded cavitands [77–79]. Our protocol works well with 2-methylresorcinol, as shown for products **1f** and **1g**, with significant yields around 80%.

Electron-deficient and halogenated 2-substitued resorcinols are notoriously difficult to engage in the cyclization reaction towards resorcin[*n*]arenes since the nucleophilic character of the attacking aromatic carbon is diminished. Specifically, there is no literature information on 2-chlororesorcinol or 2-iodoresorcinol being used in this manner, and from the few reports using 2-bromoresorcinol, it has been described to yield inseparable mixtures of oligomers [9,59]. Electron-withdrawing groups like carboxylic acids are another useful functional group instead of the halogen that provide a divergent route to other functional materials, e.g., polymers and capsules [80]. In that regard, 2,6-dihydroxybenzoic acid is known to also yield inseparable mixtures [9,81]. However, a couple of reports describe successful syntheses with reaction yields ≈40% using 2,6-dihydroxybenzoic acid and formaldehyde under basic conditions [38]. Recently, similar conditions using basic media have been employed successfully with 2-nitroresorcinol in the formation of resorcin[*n*]arenes [82–83]. We applied our protocol using 2-haloresorcinols and aliphatic aldehydes of varying lengths. Our findings indicate that chlorinated species **1h**–**j** are formed with reasonable yields ranging from 48 to 69% in 24 hours (Scheme 2). Remarkably, brominated compounds **1k**–**n** are also formed in significant yields reaching up to 94% for the *n*-pentyl-containing species **1m**. Note that species **1l** and **1m** are extensively used in the field and are prepared through the two-step synthesis described in the introduction (Scheme 1a and b) [56,58–59,63,66,80,84–88]. Furthermore, we successfully synthesized carboxylic acid-containing resorcin[4]arenes **1o**–**s** employing HFIP under an optimized reaction time of 48 hours. Compounds **1o**–**s** are reported here for the first time. Their tetracarboxylic acid head group and short-to-long aliphatic tails from ethyl to *n*-undecyl may find applications in the development of novel materials, e.g., as ligands in nanoparticle synthesis.

**Figure 1:**
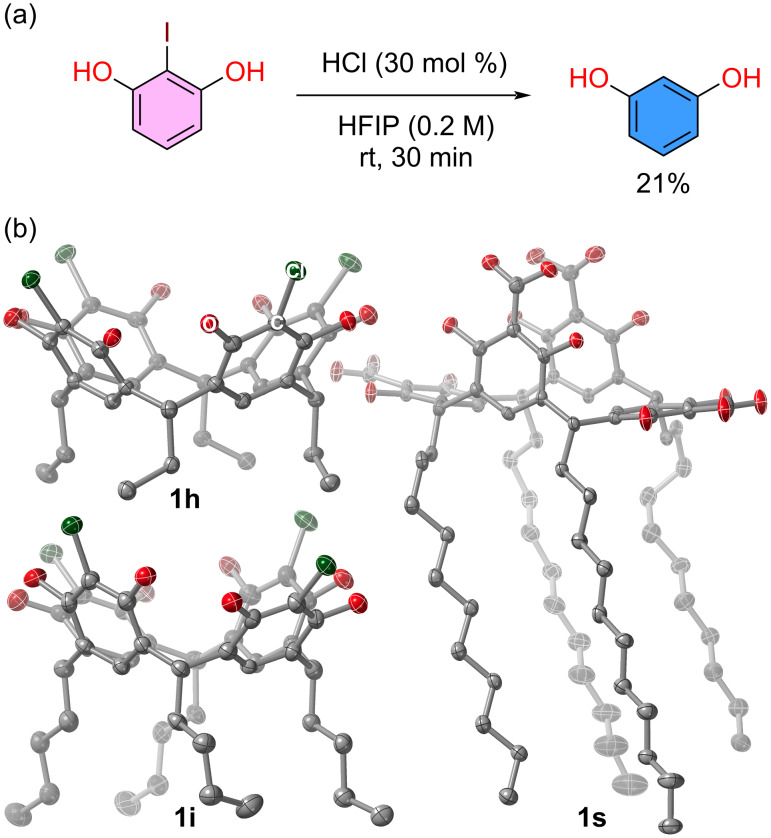
(a) Control experiment testing deiodination of 2-iodoresorcinol. (b) Molecular crystal structure of chlorinated resorcin[4]arenes **1h** and **1i**, and carboxylic acid-containing **1s** at 100 K. Thermal ellipsoids are set at 50% probability level.

We were surprised to find out that 2-iodoresorcinol did not produce the desired resorcin[*n*]arene. Repeated experiments showed resorcinol in the reaction mixture. This observation led us to run a control experiment in the absence of aldehyde, which showed that HFIP leads to metal-free deiodination of 2-iodoresorcinol (Figure 1a). Finally, while all new compounds reported herein have full spectroscopic characterization, chlorinated species **1h** and **1i**, and carboxylic acid-containing **1s**, developed high quality crystals from standing solutions in dimethyl sulfoxide for **1h** and **1i**, and methanol for **1s**. Their molecular crystal structures were determined and are shown in Figure 1b displaying the classic cone conformation for both chloro species and flattened cone or boat for **1s** [89].

Overall, access to electron-deficient and halogenated resorcin[4]arenes in one synthetic step provides building blocks to advance a wide range of chemical, physical, materials, and supramolecular applications. Future modifications of the protocol reported herein may impact the synthesis of other macrocyclic arene species, e.g., calix[*n*]arenes, calix[4]pyrroles, pillar[*n*]arenes, and cucurbit[*n*]urils [90–92].

### Perspective

Supramolecular chemistry is a mature field that has crossed boundaries into many other scientific areas. However, the work described herein exemplifies that even when protocols are well-established, a simple, yet critical modification may improve access to known species in shorter reaction times, and most importantly unveil new scaffolds that were previously inaccessible. We encourage scientists starting their careers in this area to analyze ingrained synthetic protocols towards macrocyclic arenes and challenge them as there may be many gems awaiting discovery.

## Conclusion

Introduction of HFIP to the synthesis of resorcin[*n*]arenes accelerates their reaction time significantly to under one hour for simple and commonly used starting materials, and most importantly establishes the production of new species unavailable in the past, e.g., halogenated and electron-deficient resorcin[4]arenes. Our studies suggest that the benefits of short reaction times and substrate scope obtained from the protocol developed herein may be translated to the formation of other macrocycles as long as they share a similar reaction mechanism.

## Supporting Information

File 1 Experimental procedures for reactions, and relevant spectra of all new compounds.

## Data Availability

All data that supports the findings of this study is available in the published article and/or the supporting information to this article.
