# Supplementary material for: HFIP as a versatile solvent in resorcin[n]arene synthesis
Source: Beilstein J Org Chem. 2024 Oct 2;20:2469–75. doi: 10.3762/bjoc.20.211 (PMC11457071; doi:10.3762/bjoc.20.211)
Supplement: File 1 — Experimental procedures for reactions, and relevant spectra of all new compounds. [file Beilstein_J_Org_Chem-20-2469-s001.pdf]

BEILSTEIN JOURNAL OF ORGANIC CHEMISTRY

Supporting Information

for

HFIP as a versatile solvent in resorcin[*n*]arene synthesis

Hormoz Khosravi, Valeria Stevens and Raúl Hernández Sánchez

Beilstein J. Org. Chem. **2024,** *20,* 2469–2475. doi:10.3762/bjoc.20.211

Experimental procedures for reactions, and relevant spectra of all new compounds

License and Terms: This is a supporting information file under the terms of the Creative Commons Attribution License (https://creativecommons.org/licenses/by/4.0). Please note that the reuse, redistribution and reproduction in particular requires that the author(s) and source are credited and that individual graphics may be subject to special legal provisions.
The license is subject to the *Beilstein Journal of Organic Chemistry* terms and conditions: (https://www.beilstein-journals.org/bjoc/terms)

Table of Contents

Experimental section S2

Table S1: Crystallographic data for **1h**, **1i**, and **1s**. S11

Figure S1: Conversion versus time for **1a**. S12

Figure S2: ^{1}H NMR spectrum of **1a**. S13

Figure S3: ^{1}H NMR spectrum of **1b**. S14

Figure S4: ^{1}H NMR spectrum of **1c**. S15

Figure S5: ^{1}H NMR spectrum of **1d**. S16

Figure S6: ^{1}H NMR spectrum of **1e**. S17

Figure S7: ^{1}H NMR spectrum of **1f**. S18

Figure S8: ^{1}H NMR spectrum of **1g**. S19

Figure S9: ^{1}H and ^{13}C NMR spectra of **1h**. S20

Figure S10: ^{1}H and ^{13}C NMR spectra of **1i**. S21

Figure S11: ^{1}H and ^{13}C NMR spectra of **1j**. S22

Figure S12: ^{1}H NMR spectrum of **1k**. S23

Figure S13: ^{1}H NMR spectrum of **1l**. S24

Figure S14: ^{1}H NMR spectrum of **1m**. S25

Figure S15: ^{1}H NMR spectrum of **1n**. S26

Figure S16: ^{1}H and ^{13}C NMR spectra of **1o**. S27

Figure S17: ^{1}H and ^{13}C NMR spectra of **1p**. S28

Figure S18: ^{1}H and ^{13}C NMR spectra of **1q**. S29

Figure S19: ^{1}H and ^{13}C NMR spectra of **1r**. S30

Figure S20: ^{1}H and ^{13}C NMR spectra of **1s**. S31

Figure S21: Molecular crystal structure of **1h**. S32

Figure S22: Molecular crystal structure of **1i**. S33

Figure S23: Molecular crystal structure of **1s**. S34

References S35

Experimental section

General considerations. All reactions were performed in 20 mL scintillation vials. The vials were fitted with a Teflon magnetic stirring bar and the respective caps on the vials. Gram scale reactions were performed in 100 mL round-bottomed flasks equipped with a magnetic stirring bar. Reactions were not performed under inert conditions unless otherwise noted. Hexafluoro-2-propanol was purchased from Synquest Laboratories Inc.

^{1}H and ^{13}C NMR spectra were recorded on Bruker Avance III and Bruker Avance NEO 600MHz. Chemical shifts for protons are reported in parts per million (ppm) downfield from tetramethylsilane (TMS) and are reference to residual protium in the NMR solvent (acetone: δ 2.05; MeOH: δ 3.31; $(CH_3)_2SO$: δ 2.50). Chemical shifts for carbon are reported in ppm downfield from TMS and are referenced to the carbon resonances of the solvent (acetone: δ 29.84; MeOH: δ 49.0; $(CH_3)_2SO$: δ 39.51). Data is presented as follows: chemical shift, multiplicity (s = singlet, d = doublet, t = triplet, m = multiplet, br = broad), coupling constants in hertz, and integration. Resonances corresponding to the numerous aromatic and/or aliphatic carbon atoms in the reported compounds sometimes overlap, thereby reducing the number of observed resonances.

Flow injection accurate mass analysis was conducted using an Agilent Infinity II Ultra-High Performance Liquid Chromatography (UHPLC) system interfaced to an Agilent 6545XT Quadrupole Time-of-Flight (qToF) mass spectrometer through an Agilent Jet Spray (AJS) electrospray ionization (ESI) source. The source was operated in the positive ion mode. The mobile phase composition was 30:70 0.1% formic acid in water/0.1% formic acid in acetonitrile. Injection volume for each sample was 0.5 µL.

Single crystal data for **1h**, **1i**, and **1s** were collected on a Rigaku Synergy-S diffractometer equipped with dual-beam microfocus Cu Kα and Mo Kα X-ray radiation sources paired with a Rigaku's HyPix-Arc150 detector. Temperature was maintained using an Oxford Cryosystem nitrogen flow apparatus. Single crystals suitable for X-ray structure analysis were coated with Paratone N-oil and mounted on MiTeGen Kapton loops (polyimide). Data from Rigaku's diffractometer was integrated and corrected using CrysAlisPro V42. Space group assignments were determined by examination of systematic absences, E-statistics, and successive refinement

of the structures. The program PLATON [1,2] was employed to confirm the absence of higher symmetry for any of the crystals. The positions of the heavy atoms were determined using intrinsic phasing methods using the program SHELXT[3] and SHELXL[4] with Olex2[5] interface. Successive cycles of least-square refinement followed by difference Fourier syntheses revealed the positions of the remaining non-hydrogen atoms. Non-hydrogen atoms were refined with anisotropic displacement parameters, and hydrogen atoms were added in idealized positions. Crystallographic data for **1h**, **1i**, and **1s** is given in Table S1.

Synthetic details. General procedure for the synthesis of resorcin[*n*]arenes. Compounds **1a–s** were obtained following this general reaction sequence:

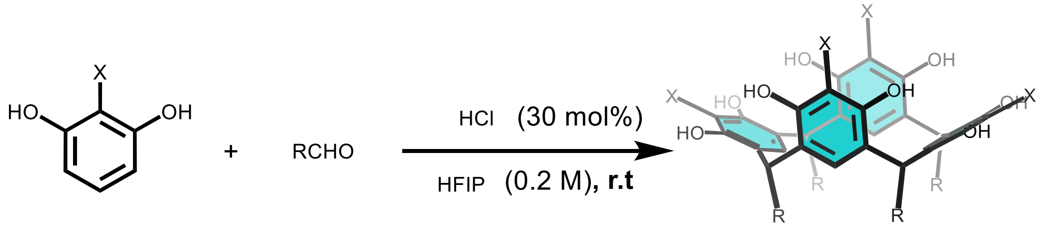

Resorcinol (1 mmol) was added to a 20 mL scintillation vial equipped with a Teflon stirring bar. Next, 5 mL of hexafluoro-2-propanol were added to the flask to create a solution. The resorcinol was left to completely dissolve for 20 minutes. Once dissolved, 1 mmol of the aldehyde was added to the solution. Subsequently, using a 9" long glass Pasteur pipette, a singular drop of 37 wt % HCl was added to the solution. A precipitate formed immediately. The reaction was stirred at room temperature for 40 minutes. Afterwards, the reaction was poured onto a filter paper in a funnel, vacuum was not applied. The filter paper was washed with 5 mL of H_2O. The precipitate was dried under high vacuum to afford a pale colored powder. **<u>**Note</u>:** *For resorcinols containing electron-withdrawing groups, the reaction must be stirred for: a) 24 h when containing halogens or b) 48 h and 50 °C when having a carboxylic acid.*

1a

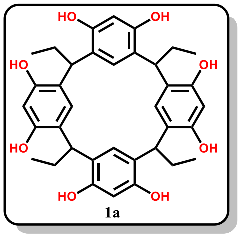

Spectroscopic signatures match literature reports.[6]

^{1}H NMR (600 MHz, Acetone-*d$_6$*): δ_H = 8.33 (s, 8H), 7.42 (s, 4H), 6.11 (s, 4H), 4.05 (t, J = 7.9 Hz, 4H), 2.19 (p, J = 7.4 Hz, 8H), and 0.77 (t, J = 7.2 Hz, 12H) ppm.

1b

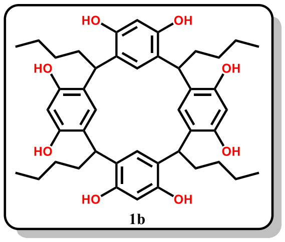

Spectroscopic signatures match literature reports.[7]

Pale beige-yellow colored solid, 179.0 mg (92% yield).

^{1}H NMR (600 MHz, Acetone-*d$_6$*): δ_H = 8.43 (s, 8H), 7.56 (s, 4H), 6.24 (s, 4H), 4.30 (td, J = 7.9, 1.8 Hz, 4H), 2.30 (q, J = 7.7 Hz, 8H), 1.42 – 1.35 (m, 8H), 1.31 – 1.25 (m, 8H), and 0.89 (t, J = 7.3 Hz, 12H) ppm.

1c

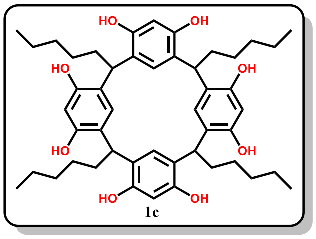

Spectroscopic signatures match literature reports.[8]

Pale yellow solid, 201.4 mg (98% yield).

^{1}H NMR (500 MHz, Acetone-*d$_6$*): δ_H = 8.45 (s, 8H), 7.56 (s, 4H), 6.24 (s, 4H), 4.31 (t, J = 7.9 Hz, 4H), 2.30 (q, J = 7.6 Hz, 8H), 1.37 – 1.31 (m, 24H), and 0.90 (d, J = 7.0 Hz, 12H) ppm.

1d

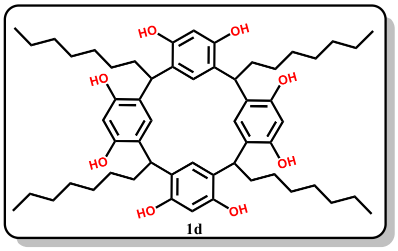

Spectroscopic signatures match literature reports.[9]

Pale yellow solid, 210.0 mg (66% yield).

^{1}H NMR (600 MHz, Methanol-*d$_4$*): δ_H = 7.10 (s, 4H), 6.13 (s, 4H), 4.18 (t, J = 7.9 Hz, 4H), 2.08 (q, J = 7.8 Hz, 8H), 1.26 – 1.20 (m, 40H), and 0.84 (t, J = 7.0 Hz, 12H) ppm.

1e

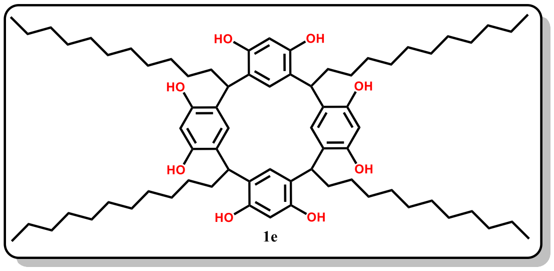

Spectroscopic signatures match literature reports.[6]

Pale yellow solid, 170.1 mg (62% yield).

^{1}H NMR (500 MHz, Acetone-*d$_6$*): δ_H = 8.44 (s, 8H), 7.55 (s, 4H), 6.24 (s, 4H), 4.31 (t, J = 7.9 Hz, 4H), 2.30 (q, J = 7.7 Hz, 8H), 1.39 (s, 72H), and 0.89 (s, 12H) ppm.

1f

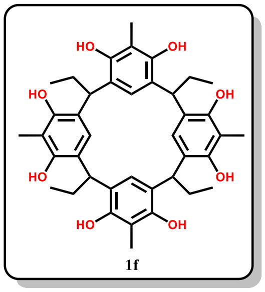

Spectroscopic signatures match literature reports.[10]

To obtain the pure compound, the solid that was initially collected through gravity filtration was slightly dissolved in acetone. A dropwise addition of hexanes was added to precipitate out the pure compound. The precipitate that occurs after the addition of hexanes is then collected via gravity filtration. Brown solid, 139.7 mg (84% yield).

^{1}H NMR (600 MHz, Methanol-*d$_4$*): δ_H = 7.09 (s, 4H), 4.25 (t, J = 7.9 Hz, 4H), 2.20 (p, J = 7.3 Hz, 8H), 2.03 (s, 12H), and 0.91 (t, J = 7.2 Hz, 12H) ppm.

1g

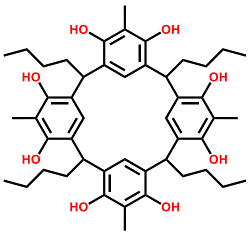

1g

Spectroscopic signatures match literature reports.[10]

Light beige-yellow solid, 159.0 mg (81% yield).

^{1}H NMR (600 MHz, Acetone-*d$_6$*): δ_H = 7.92 (s, 8H), 7.41 (s, 4H), 4.38 – 4.34 (m, 4H), 2.27 (q, J = 7.7 Hz, 8H), 2.03 (s, 12H), 1.37 (q, J = 7.2 Hz, 8H), 1.27 – 1.23 (m, 8H), and 0.87 (t, J = 7.3 Hz, 12H) ppm.

1h

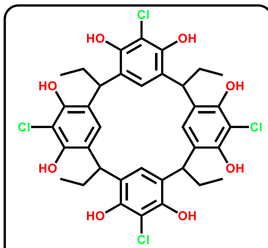

1h

To obtain the pure compound, the solid that was collected through gravity filtration was slightly dissolved in acetone. A dropwise addition of hexanes was added to precipitate out the pure compound. The precipitate that occurs after the addition of hexanes is then collected via gravity filtration. White solid, 89.1 mg (48% yield).

^{1}H NMR (500 MHz, DMSO-*d$_6$*): δ_H = 8.95 (s, 8H), 7.18 (s, 4H), 4.30 (t, J = 7.9 Hz, 4H), 2.09 (d, J = 2.9 Hz, 8H), and 0.81 (t, J = 7.2 Hz, 12H) ppm;**^{13}C NMR (126 MHz, DMSO-*d6*)**: δ_C = 147.9, 124.5, 123.1, 109.1, 36.9, 27.0, and 12.4 ppm; **HRMS-ESI** (m/z): calculated for $C_{36}H_{36}Cl_4O_8$ $[M+H]^+$ 737.1237 found 737.1240.

1i

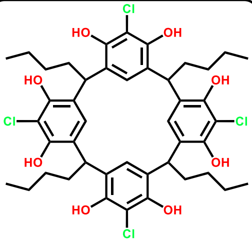

1i

To obtain the pure compound, the solid that was initially collected through gravity filtration was slightly dissolved in acetone. It was then stored in the freezer for 10 minutes. A dropwise addition of hexanes was added to precipitate out the pure compound. The precipitate that occurs after the addition of hexanes is then collected via gravity filtration. White solid, 148.5 mg (69% yield).

^{1}H NMR (500 MHz, DMSO-*d$_6$*): δ_H = 8.85 (s, 8H), 7.07 (s, 4H), 4.43 (t, J = 7.8 Hz, 4H), 1.99 (q, J = 7.7 Hz, 8H), 1.30 (p, J = 7.5 Hz, 8H), 1.16 (ddt, J = 15.2, 9.8, 6.1 Hz, 8H), and 0.84 (t, J = 7.3 Hz, 12H) ppm; **^{13}C NMR (126 MHz, DMSO-*d6*)**: δ_H = 147.9, 124.4, 123.2, 109.2, 35.0, 34.0, 29.9, 22.2, and 14.1 ppm; **HRMS-ESI** (m/z): calculated for $C_{44}H_{52}Cl_4O_8$ $[M+H]^+$ 849.2489 found 849.2492.

1j

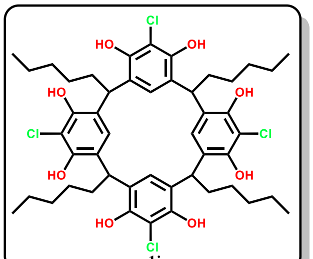

1j

To obtain the pure compound, the solid that was collected through gravity filtration was slightly dissolved in acetone. It was then stored in the freezer for 10 minutes. A dropwise addition of MeOH/H_2O (1:1) was added to precipitate out the pure compound. The precipitate that occurs after the addition of MeOH/H_2O is then collected via gravity filtration. White solid, 122.2 mg (52% yield).

^{1}H NMR (500 MHz, DMSO-*d₆*): δ_H = 8.85 (s, 8H), 7.06 (s, 4H), 4.43 (t, J = 7.7 Hz, 4H), 1.97 (q, J = 7.7 Hz, 8H), 1.24 (ddd, J = 26.3, 15.6, 10.7 Hz, 24H), and 0.82 (t, J = 6.9 Hz, 12H) ppm; **^{13}C NMR (151 MHz, DMSO-*d₆*)**: δ_c = 148.3, 124.9, 123.6, 109.6, 35.5, 34.7, 31.9, 27.8, 22.7, and 14.4 ppm; **HRMS-ESI** (m/z): calculated for $C_{48}H_{60}Cl_4O_8$ $[M+H]^+$ 905.3115 found 905.3114.

1k

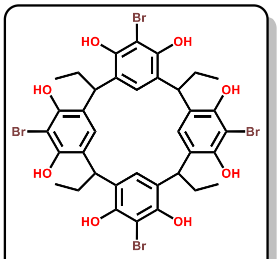

1k

Spectroscopic signatures match literature reports.[11]

To obtain the pure compound, the solid that was initially collected through gravity filtration was boiled in acetone. It was then stored in the freezer for 10 min. A dropwise addition of hexanes was added to precipitate out the pure compound. The precipitate that occurs after the addition of hexanes is then collected via gravity filtration. White solid, 73.5 mg (31% yield).

^{1}H NMR (500 MHz, DMSO-*d₆*): δ_H = 9.15 (s, 8H), 7.43 (s, 4H), 4.23 (t, J = 7.9 Hz, 4H), 2.25 (p, J = 7.3 Hz, 8H), and 0.83 (t, J = 7.2 Hz, 12H) ppm.

1l

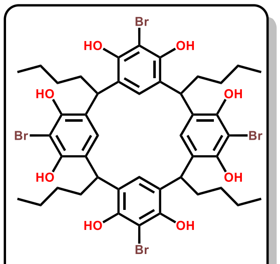

1l

Spectroscopic signatures match literature reports.[12]

To obtain the pure compound, the solid that was initially collected through gravity filtration was boiled in acetone. It was then stored in the freezer for 10 min. A dropwise addition of hexanes was added to precipitate out the pure compound. The precipitate that occurs after the addition of hexanes is then collected via gravity filtration. White solid, 168.9 mg (61% yield).

^{1}H NMR (600 MHz, DMSO-*d₆*): δ_H = 9.09 (s, 8H), 7.36 (s, 4H), 4.34 (t, J = 7.9 Hz, 4H), 2.18 (q, J = 7.7 Hz, 8H), 1.34 (h, J = 7.4 Hz, 8H), 1.21 – 1.13 (m, 8H), and 0.85 (t, J = 7.3 Hz, 12H) ppm.

1m

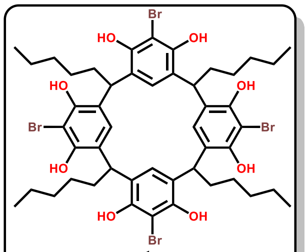

Spectroscopic signatures match literature reports.[13]

To obtain the pure compound, the solid that was initially collected through gravity filtration was boiled in acetone. It was then stored in the freezer for 10 minutes. A dropwise addition of a (1:1:1) mixture of hexanes, MeOH, water was added. The precipitate that occurs after the addition of the previous solvent mixture is then collected via gravity filtration. Light brown solid, 263.7 mg (94% yield).

^{1}H NMR (600 MHz, DMSO-*d$_6$*): δ_H = 9.14 (s, 8H), 7.40 (s, 4H), 4.41 (t, J = 7.8 Hz, 4H), 2.22 (q, J = 7.7 Hz, 8H), 1.39 – 1.35 (m, 8H), 1.33 – 1.29 (m, 8H), 1.26 – 1.22 (m, 8H), and 0.90 (t, J = 7.1 Hz, 12H) ppm.

1n

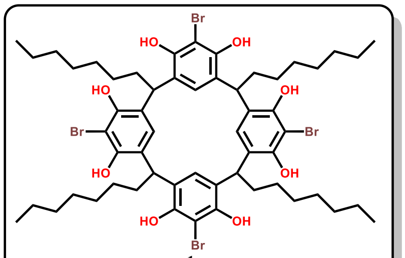

To obtain the pure compound, the solid that was initially collected through gravity filtration was boiled in acetone. It was then stored in the freezer for 10 minutes. A dropwise addition of hexanes, water, and MeOH (1:1:1) was added to precipitate out the pure compound. The precipitate that occurs after the addition of the previous solvent mixture is then collected via gravity filtration. Brown solid, 129.8 mg (42% yield).

^{1}H NMR (600 MHz, DMSO-*d$_6$*): δ_H = 9.06 (s, 8H), 7.32 (s, 4H), 4.35 (t, J = 7.8 Hz, 4H), 2.14 (q, J = 7.7 Hz, 8H), 1.31 (q, J = 7.2 Hz, 8H), 1.23 (dt, J = 10.2, 5.3 Hz, 24H), 1.18 – 1.16 (m, 8H), and 0.84 (t, J = 7.1 Hz, 12H) ppm; **^{13}C NMR (151 MHz, DMSO-*d$_6$*)**: δ_C = 148.7, 125.5, 123.8, 101.4, 35.5, 33.7, 31.4, 29.2, 29.0, 27.8, 22.2, and 14.0 ppm; **HRMS-ESI** (m/z): calculated for $C_{56}H_{76}Br_4O_8$ $[M+H]^+$ 1193.2346 found 1193.2346.

1o

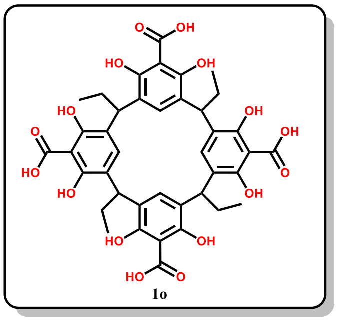

To obtain the pure compound, the solvent was evacuated from the scintillation vial. Next, water is added to the solid and allowed to stir for 1 hour. The precipitate is then collected through gravity filtration. Beige solid, 226.7 mg (69% yield).

^{1}H NMR (600 MHz, DMSO-*d$_6$*): δ_H = 9.55 (s, 12H), 7.11 (s, 4H), 4.51 (t, J = 7.7 Hz, 4H), 1.89 (p, J = 7.3 Hz, 8H), and 0.86 (t, J = 7.2 Hz, 12H) ppm; **^{13}C NMR (151 MHz, DMSO-*d$_6$*)**: δ_C = 173.7, 156.7, 131.8, 121.4, 100.3, 35.8, 28.1, and 13.1 ppm; **HRMS-ESI** (m/z): calc. for $C_{40}H_{40}O_{16}$ $[M+H]^+$ 777.2389 found 777.2383.

1p

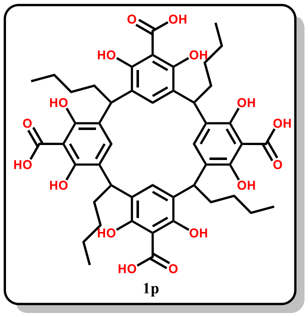

To obtain the pure compound, the solvent was evacuated from the scintillation vial. Next, water is added to the solid and allowed to stir for 1 hour. The precipitate is then collected through gravity filtration. Beige solid, 134.2 mg (60% yield).

^{1}H NMR (600 MHz, Acetone-*d$_6$*): δ_H = 10.36 (s, 12H), 7.22 (s, 4H), 4.67 (t, J = 7.6 Hz, 4H), 1.95 (q, J = 7.4 Hz, 8H), 1.41 – 1.34 (m, 8H), 1.33 – 1.27 (m, 8H), and 0.88 (t, J = 7.2 Hz, 12H) ppm; **^{13}C NMR (151 MHz, Acetone-*d$_6$*)**: δ_C = 172.7, 157.6, 133.8, 123.3, 100.2, 35.6, 35.1, 31.4, 23.7, and 14.7 ppm; **HRMS-ESI** (m/z): calculated for $C_{48}H_{56}O_{16}$ $[M+H]^+$ 889.3641 found 889.3621.

1q

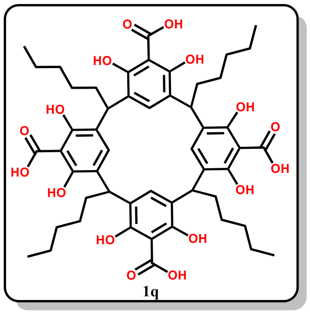

To obtain the pure compound, the solvent was evacuated from the scintillation vial. Next, water is added to the solid and allowed to stir for 1 hour. The precipitate is then collected through gravity filtration. Beige solid, 223.0 mg (92% yield).

^{1}H NMR (600 MHz, DMSO-*d$_6$*): δ_H = 10.13 (s, 12H), 7.04 (s, 4H), 4.58 (t, J = 7.6 Hz, 4H), 1.82 (q, J = 7.6 Hz, 8H), 1.31 – 1.21 (m, 24H), and 0.89 – 0.84 (m, 12H) ppm; **^{13}C NMR (151 MHz, DMSO-*d$_6$*)**: δ_C = 173.7, 156.6, 131.7, 121.3, 100.4, 35.1, 34.1, 32.0, 27.9, 22.6, and 14.4 ppm; **HRMS-ESI** (m/z): calculated for $C_{52}H_{64}O_{16}$ $[M+H]^+$ 945.4267 found 945.4275.

1r

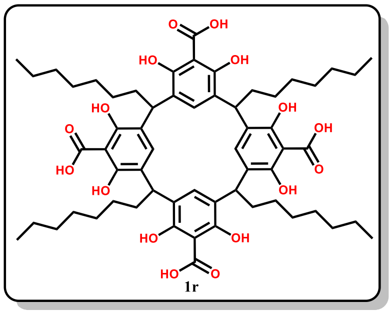

To obtain the pure compound, the solvent was evacuated from the scintillation vial. Next, water is added to the solid and allowed to stir for 1 hour. The precipitate is then collected through gravity filtration. Beige solid, 264.8 mg (64% yield).

^{1}H NMR (500 MHz, DMSO-*d$_6$*): δ_H = 10.79 (s, 12H), 6.91 (s, 4H), 4.47 (t, J = 7.6 Hz, 4H), 1.68 (q, J = 7.5 Hz, 8H), 1.13 (dt, J = 23.5, 8.9 Hz, 40H), and 0.74 (t, J = 6.9 Hz, 12H) ppm; **^{13}C NMR (126 MHz, DMSO-*d$_6$*)**: δ_C = 173.6, 156.6, 131.6, 121.4, 100.3, 35.2, 34.1, 31.8, 29.7, 29.2, 28.2, 22.5, and 14.3 ppm; **HRMS-ESI** (m/z): calculated for $C_{60}H_{80}O_{16}$ $[M+H]^+$ 1057.5520 found 1057.5526.

1s

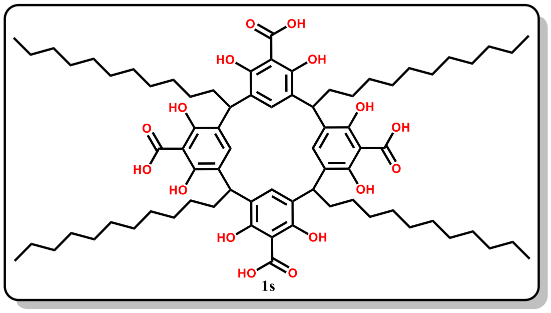

To obtain the pure compound, the solvent was evacuated from the scintillation vial. Next, water is added to the solid and allowed to stir for 1 hour. The precipitate is then collected through gravity filtration. Beige solid, 320.6 mg (95% yield).

^{1}H NMR (600 MHz, Acetone-*d$_6$*): δ_H = 10.55 (s, 12H), 7.09 (s, 4H), 4.55 (t, J = 7.6 Hz, 4H), 1.82 (q, J = 7.3 Hz, 8H), 1.18 (d, J = 45.3 Hz, 72H), and 0.74 (t, J = 6.9 Hz, 12H) ppm; **^{13}C NMR (151 MHz, Acetone-*d$_6$*)**: δ_C = 171.7, 156.5, 132.5, 122.2, 99.2, 34.5, 34.3, 31.8, 29.7, 29.7, 29.6, 29.6, 29.6, 29.3, 28.0, 22.5, and 13.5 ppm; **HRMS-ESI** (m/z): calculated for $C_{76}H_{112}O_{16}$ $[M–H]^–$ 1279.7874 found 1279.7859.

Table S1. Crystallographic data for **1h** and **1i**.

	**1h**	**1i**	**1s**
**CCDC Number**	2365251	2365250	2380394
**Chemical formula**	$C_{36}H_{36}Cl_4O_8$ ·4(C_2H_6OS)	$C_{44}H_{52}Cl_4O_8$ ·4(C_2H_6OS)	2($C_{76}H_{112}O_{16}$) ·15(CH_4O)
**Formula weight**	1050.96	1163.16	3043.93
**Space group**	$P2_1/c$	*P–1*	*P–1*
a **(Å)**	13.9271 (3)	13.0540 (4)	13.8306 (1)
b **(Å)**	13.6605 (3)	13.1859 (4)	25.2831 (2)
c **(Å)**	25.9533 (5)	17.8437 (7)	27.2500 (4)
α **(deg)**	90	83.153 (3)	103.911 (1)
β **(deg)**	96.411 (2)	85.142 (3)	91.044 (1)
γ **(deg)**	90	72.146 (2)	99.080 (1)
V **($Å^3$)**	4906.77 (18)	2898.88 (17)	9117.72 (17)
Z	4	2	2
**μ (mm^{-1})**	4.28	3.67	0.65
**T (K)**	100	100	100
**$R1^a$ ($wR2^b$)**	0.099 (0.279)	0.199 (0.594)	0.166 (0.518)
**Reflections**	10143	10640	34316
**Radiation type**	Cu $K\alpha$	Cu $K\alpha$	Cu $K\alpha$

$^aR1 = [\Sigma w(F_o - F_c)^2/\Sigma wF_o^2]^{1/2}$; $^bwR2 = [\Sigma[w(F_o^2 - F_c^2)^2]/\Sigma w(F_o^2)^2]^{1/2}]$, $w = 1/[\sigma^2(F_o^2) + (aP)^2 + bP]$, where $P = [\max(F_o^2,0) + 2(F_c^2)]/3$

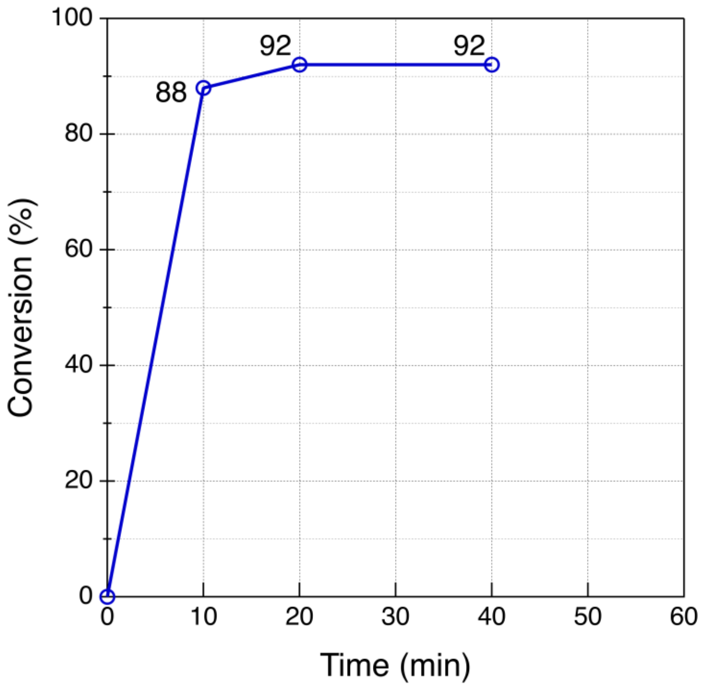

Figure S1. Conversion versus time for **1a** following conditions of Table 1, entry 2.

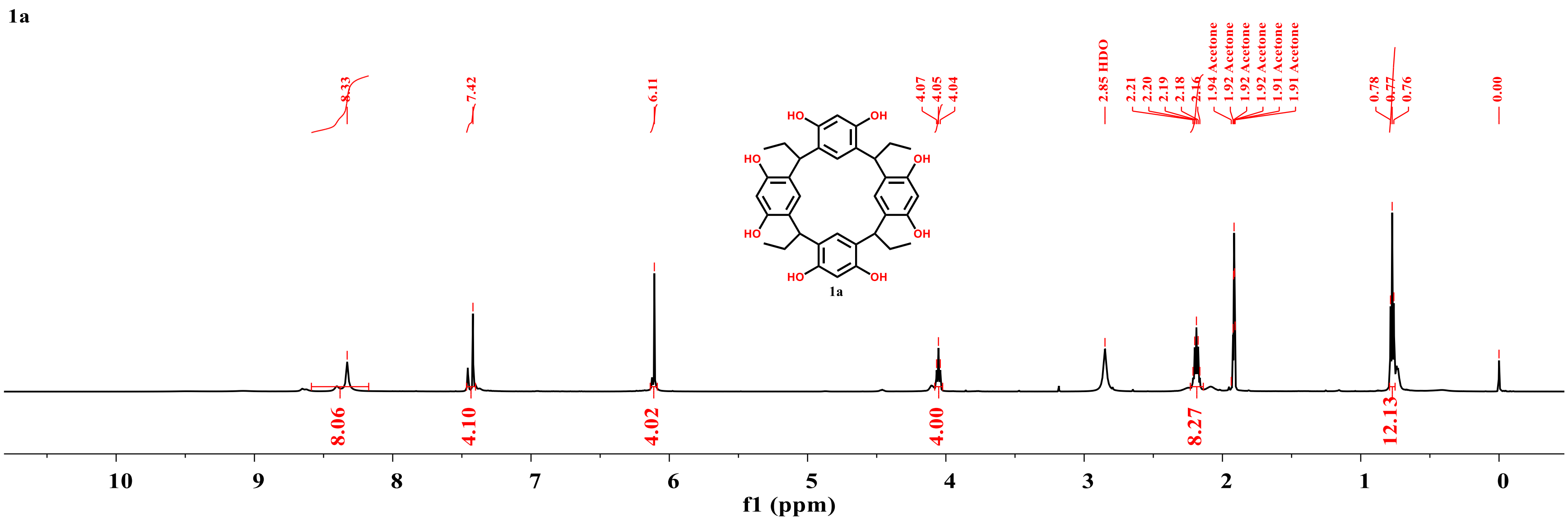

Figure S2. ^{1}H NMR spectrum (600 MHz) of compound **1a**. Data collected in acetone-d_6 at 20 °C.

1b

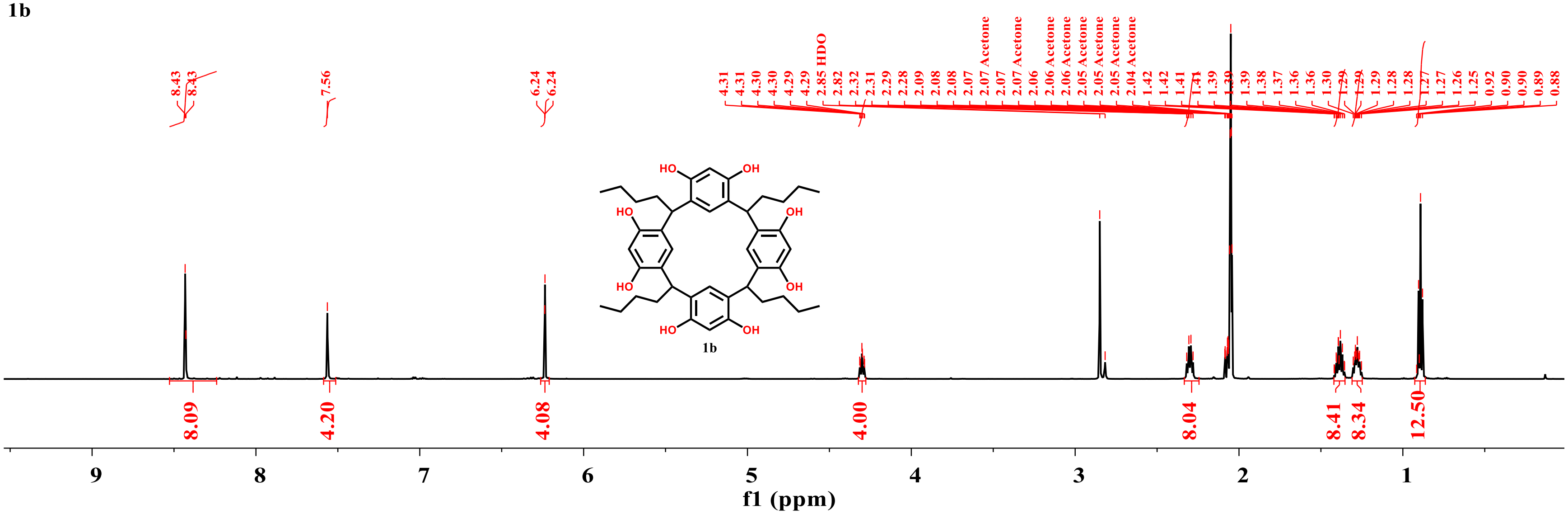

Figure S3. ^{1}H NMR spectrum (600 MHz) of compound **1b**. Data collected in acetone-d_6 at 20 °C.

1c

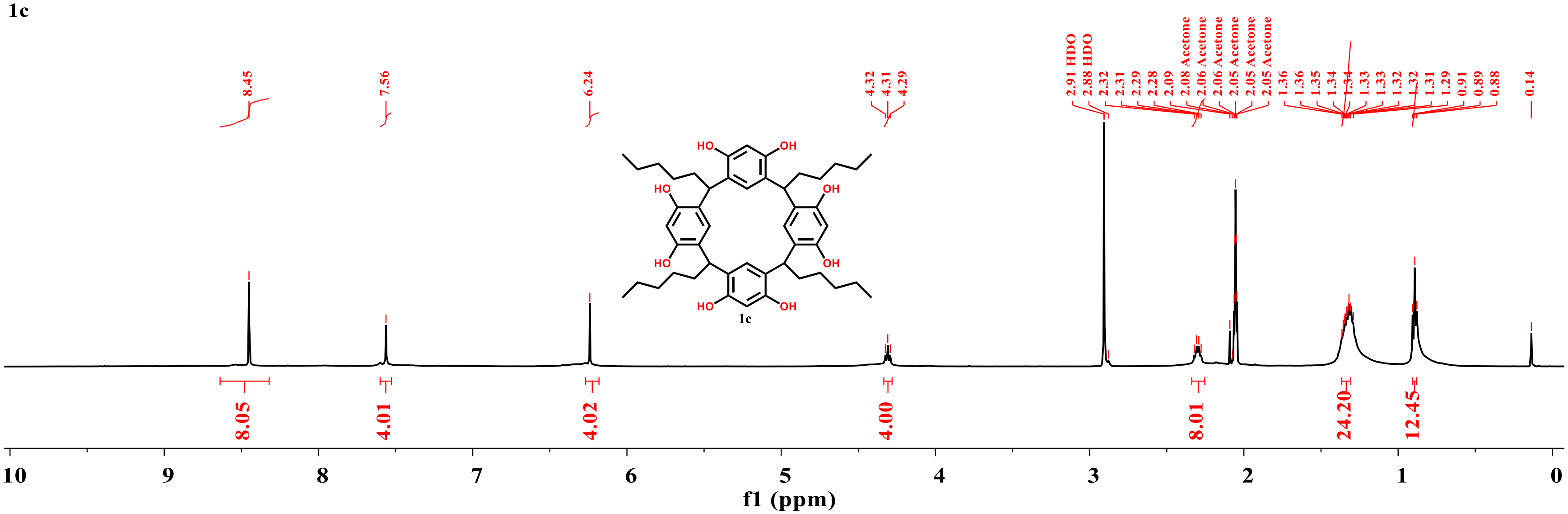

Figure S4. ^{1}H NMR spectrum (600 MHz) of compound **1c**. Data collected in acetone-d_6 at 20 °C.

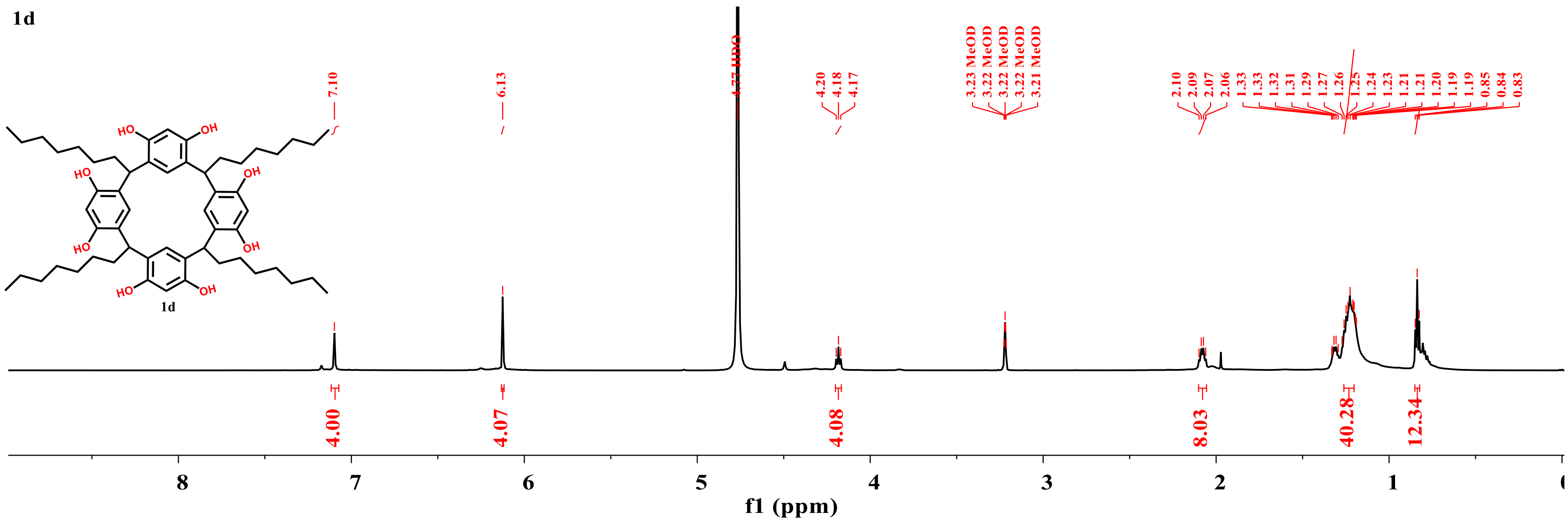

Figure S5. ^{1}H NMR spectrum (600 MHz) of compound **1d**. Data collected in methanol-d_4 at 20 °C.

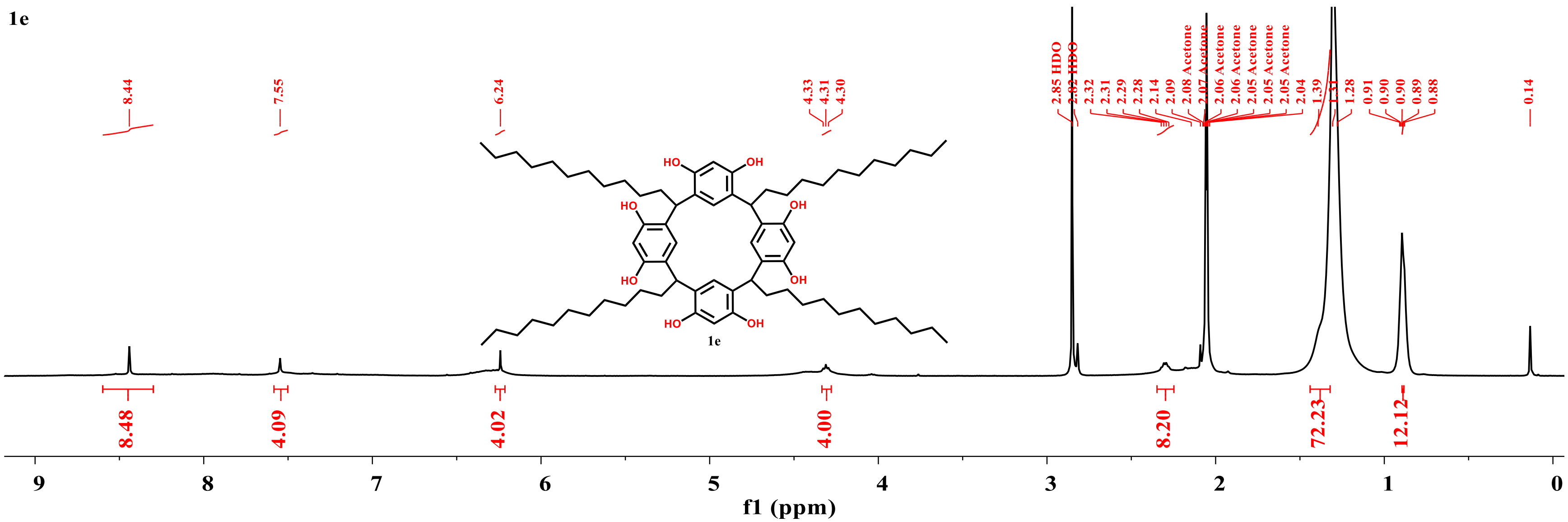

Figure S6. ^{1}H NMR spectrum (500 MHz) of compound **1e**. Data collected in acetone-d_6 at 20 °C.

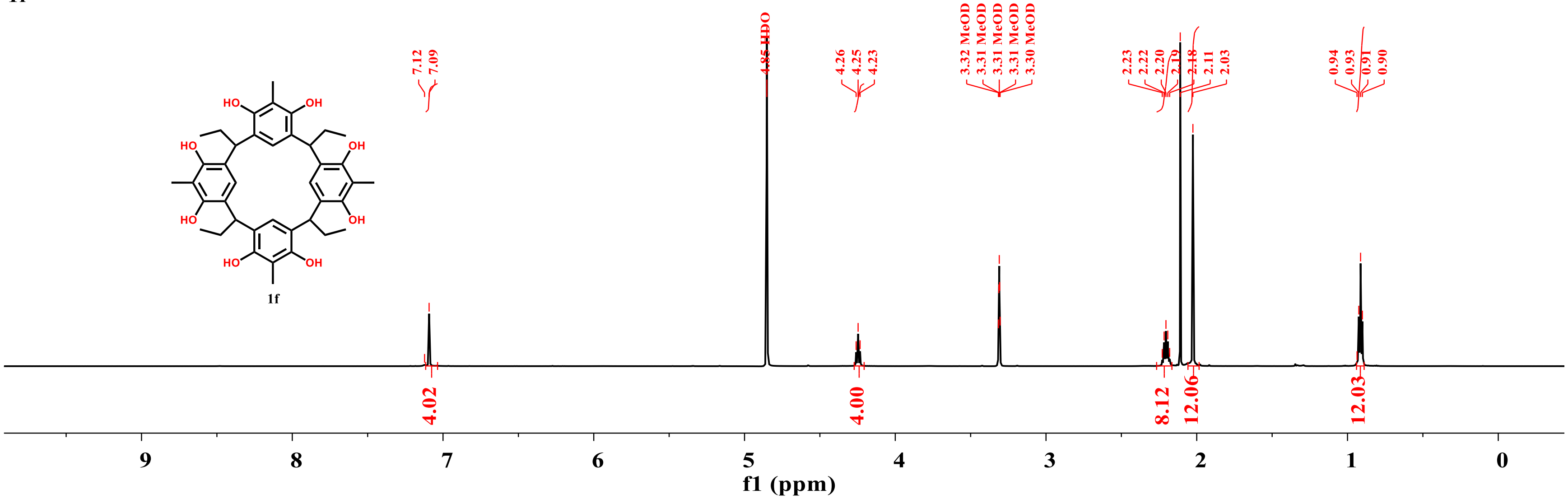

Figure S7. ^{1}H NMR spectrum (600 MHz) of compound **1f**. Data collected in methanol-d_4 at 20 °C.

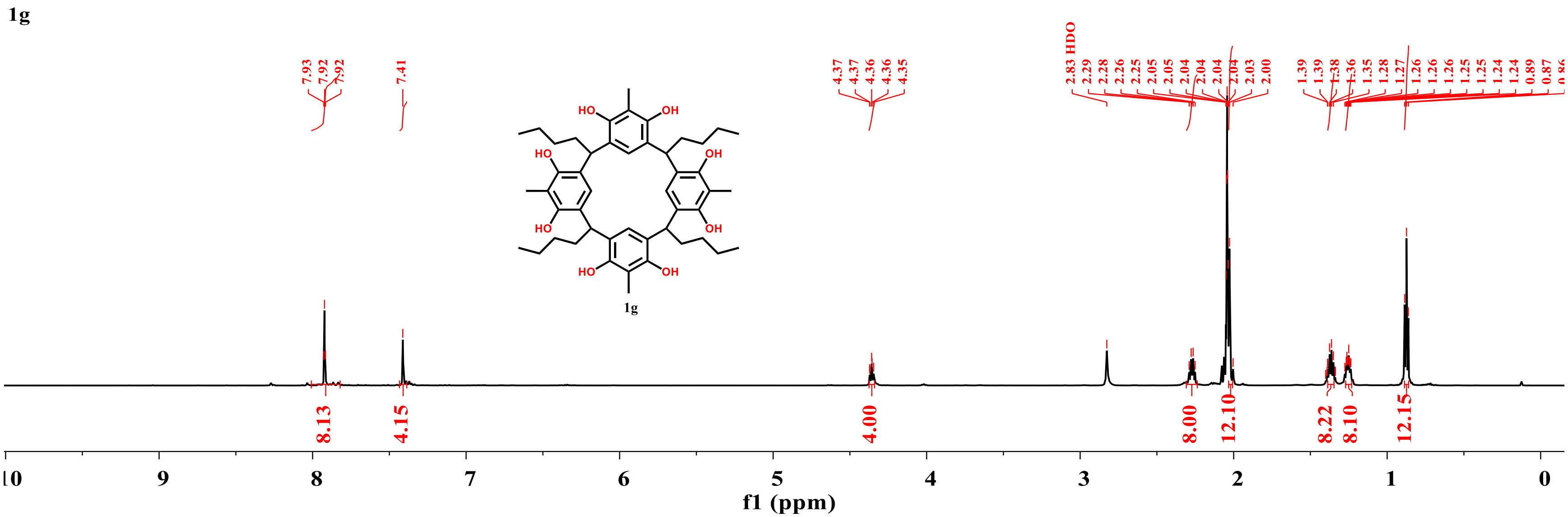

Figure S8. ^{1}H NMR spectrum (600 MHz) of compound **1g**. Data collected in acetone-d_6 at 20 °C.

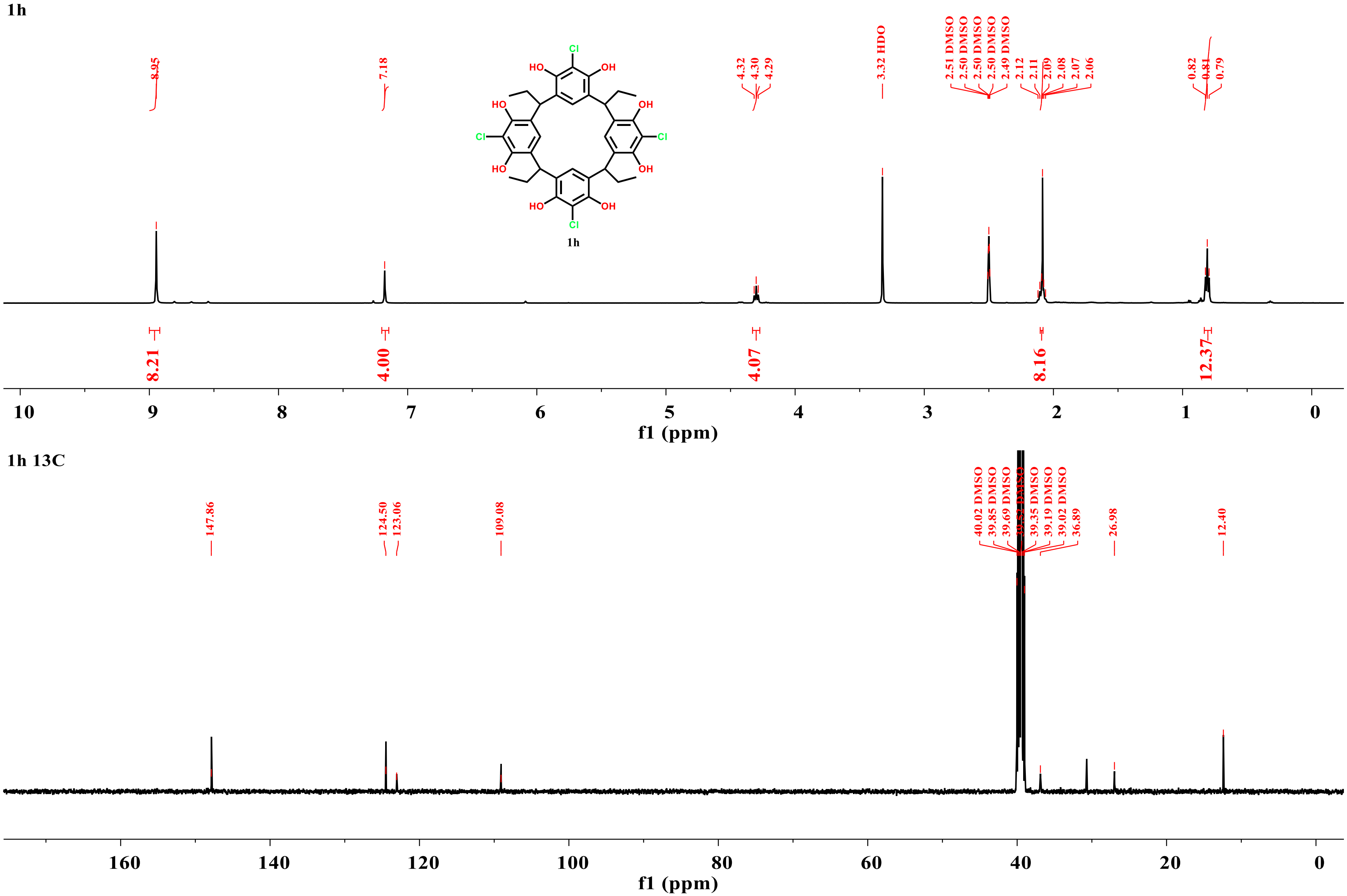

Figure S9. ^{1}H and ^{13}C NMR spectra of compound **1h**. Data collected in DMSO-d_6 at 20 °C.

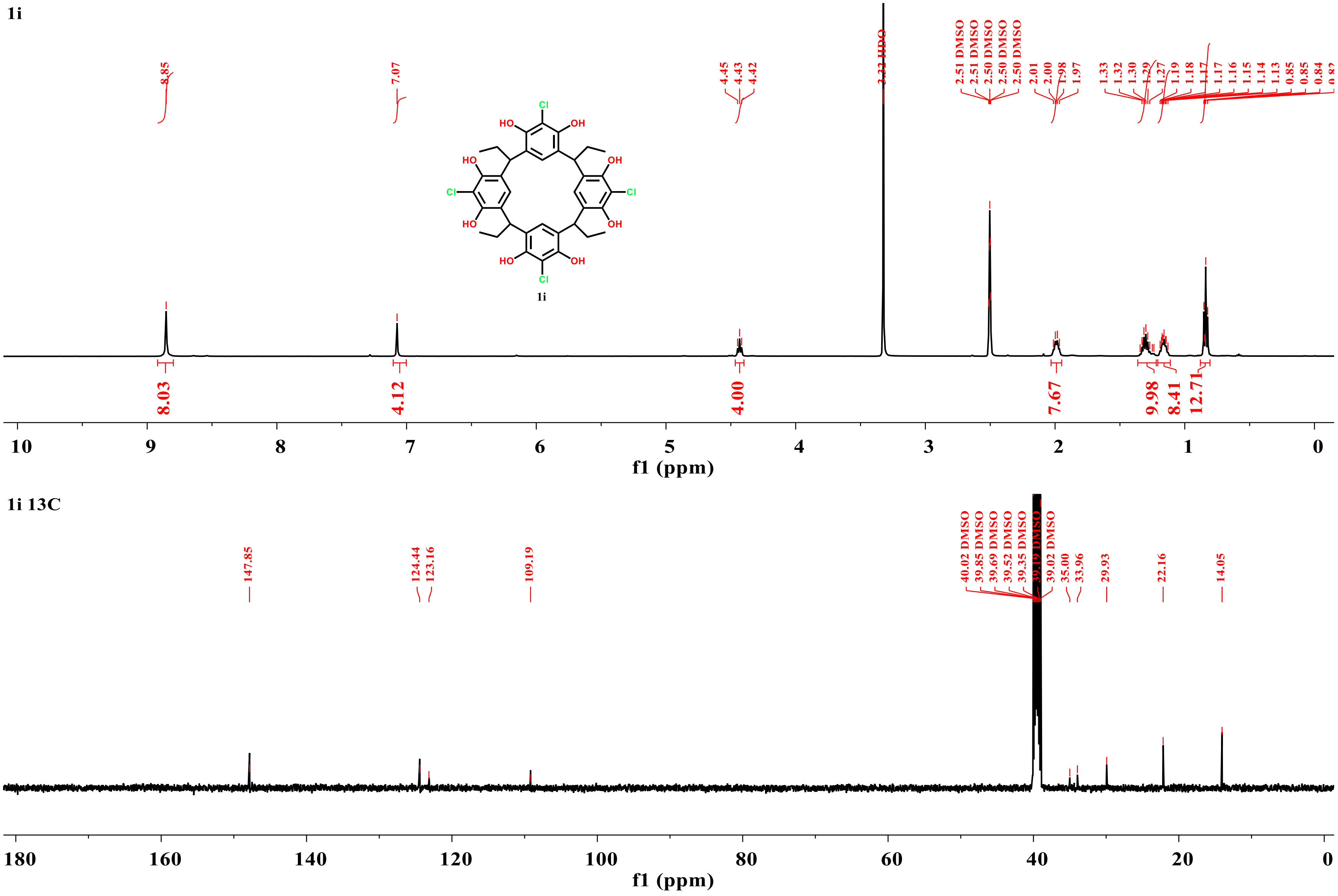

Figure S10. ^{1}H and ^{13}C NMR spectra of compound **1i**. Data collected in DMSO-d_6 at 20 °C.

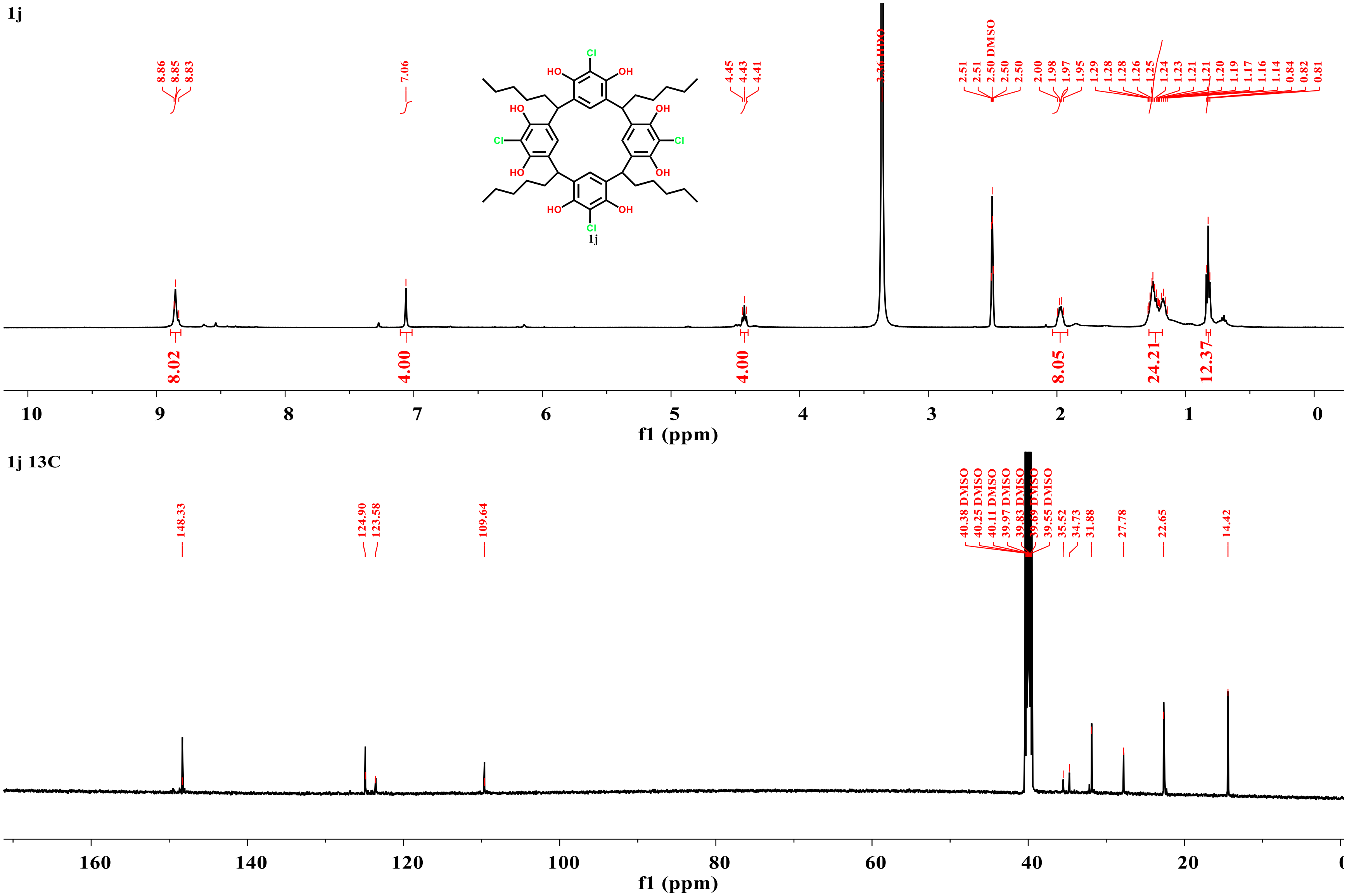

Figure S11. ^{1}H and ^{13}C NMR spectra of compound **1j**. Data collected in DMSO-d_6 at 20 °C.

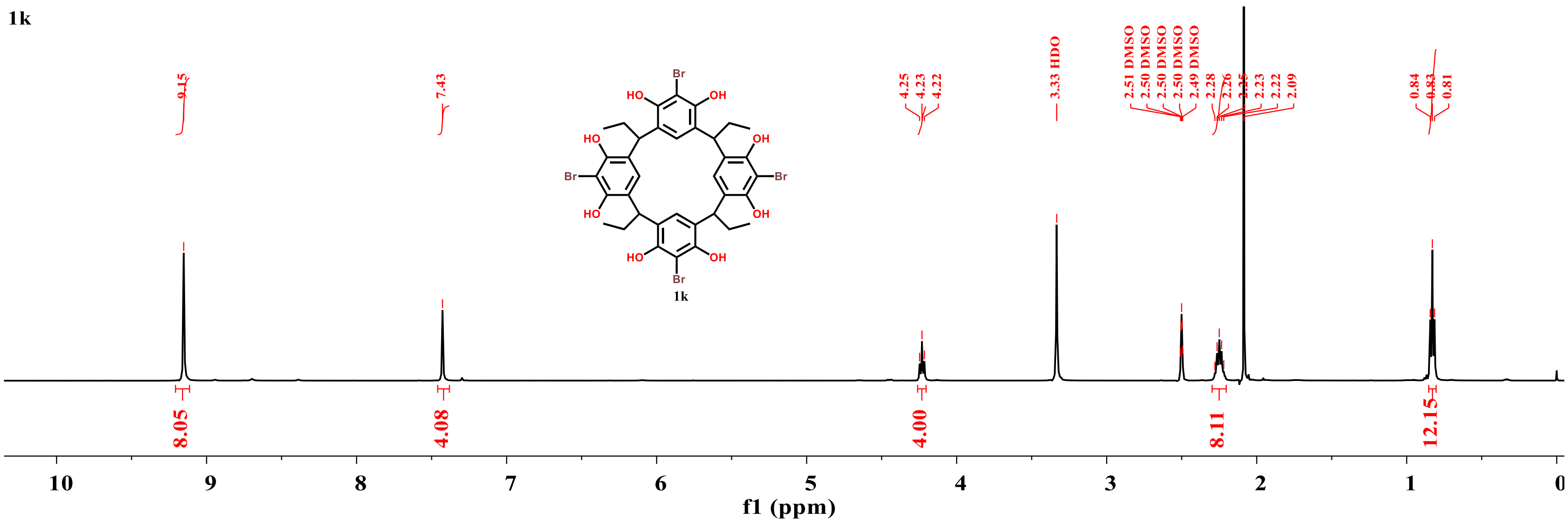

Figure S12. ^{1}H NMR spectrum (500 MHz) of compound **1k**. Data collected in DMSO-d_6 at 20 °C.

1l

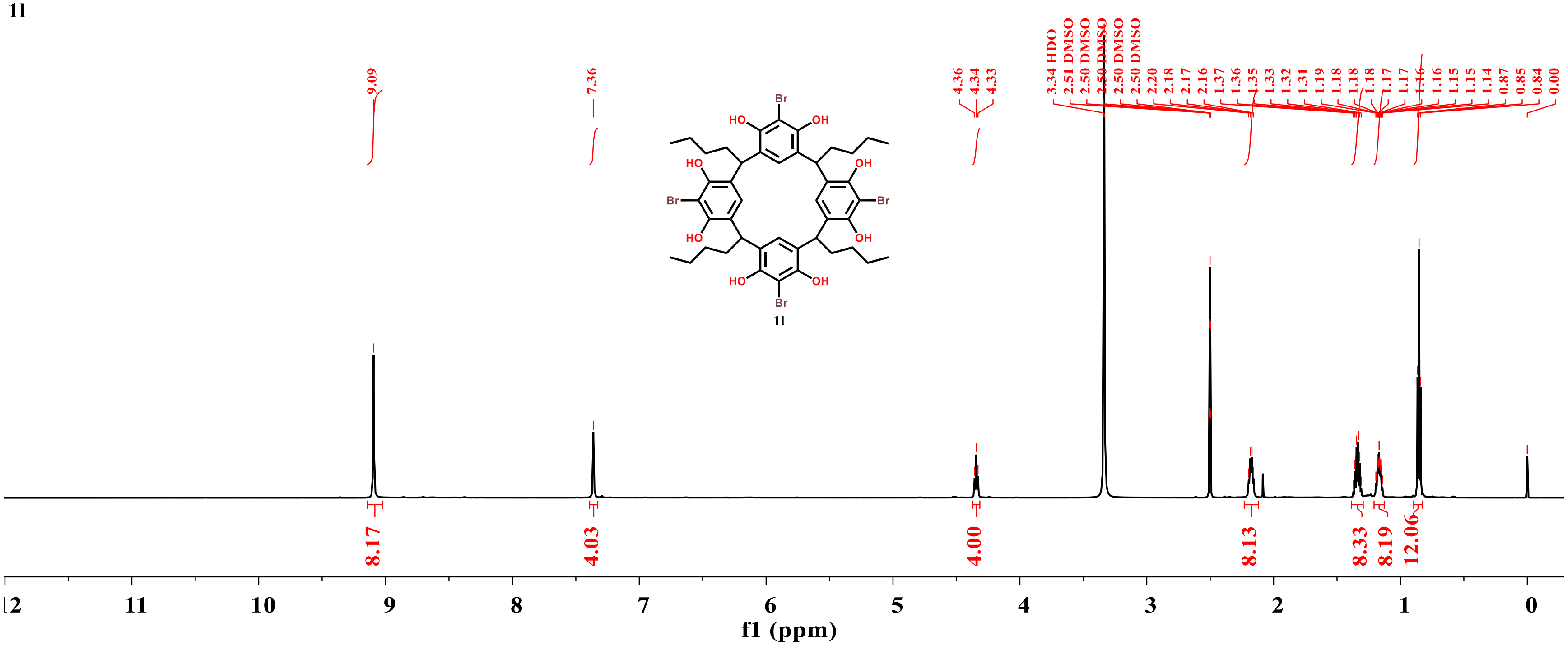

Figure S13. ^{1}H NMR spectrum (600 MHz) of compound **1l**. Data collected in DMSO-d_6 at 20 °C.

1m

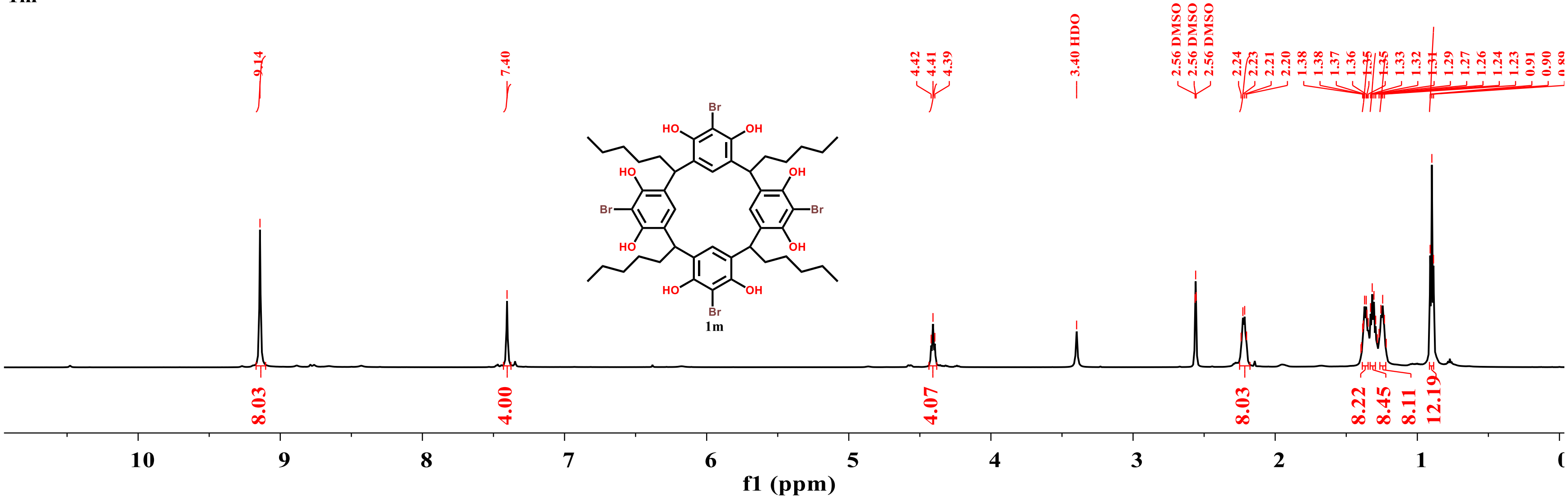

Figure S14. ^{1}H NMR spectrum (600 MHz) of compound **1m**. Data collected in DMSO-d_6 at 20 °C.

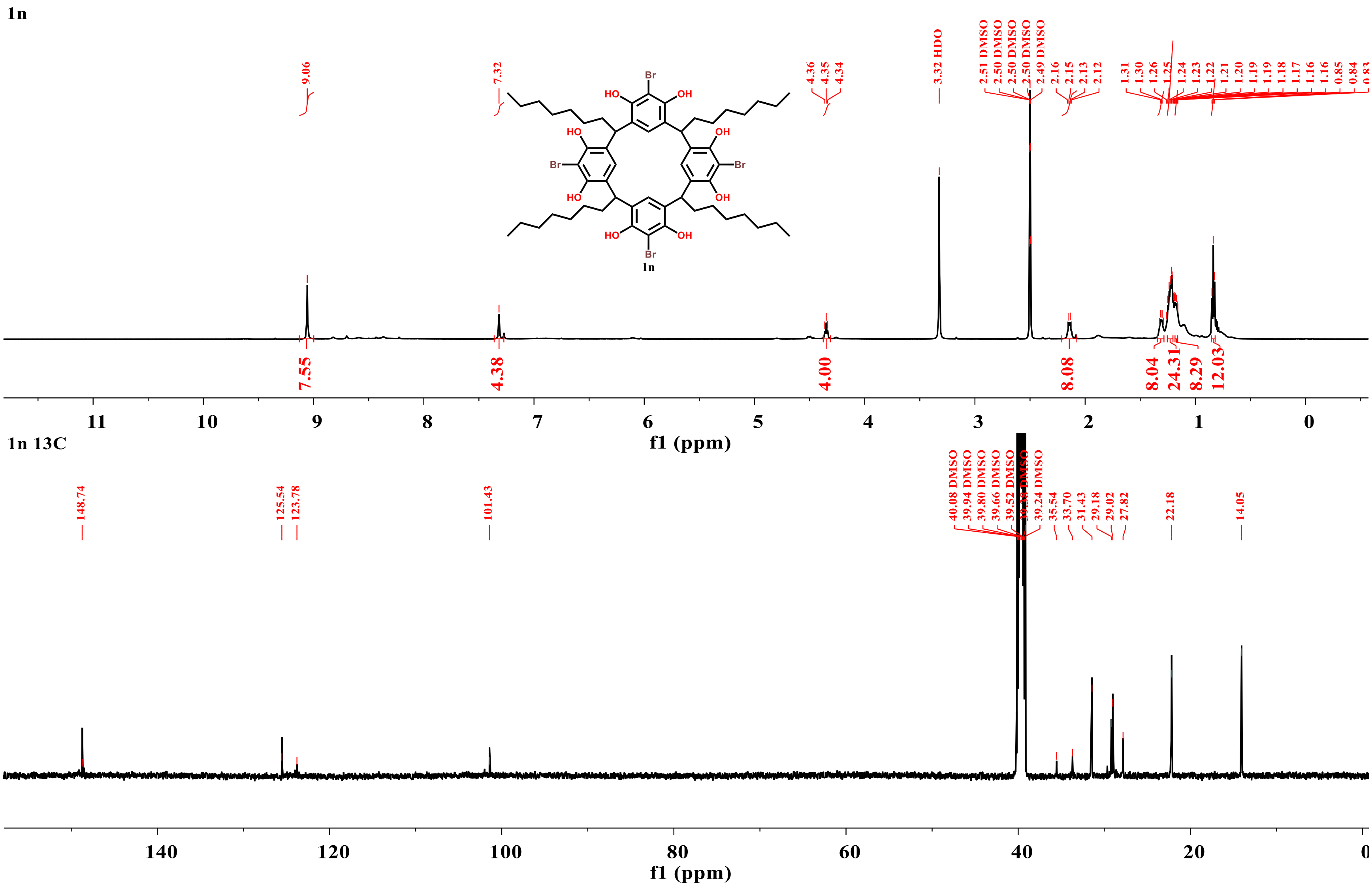

Figure S15. ^{1}H and ^{13}C NMR spectra of compound **1n**. Data collected in DMSO-d_6 at 20 °C.

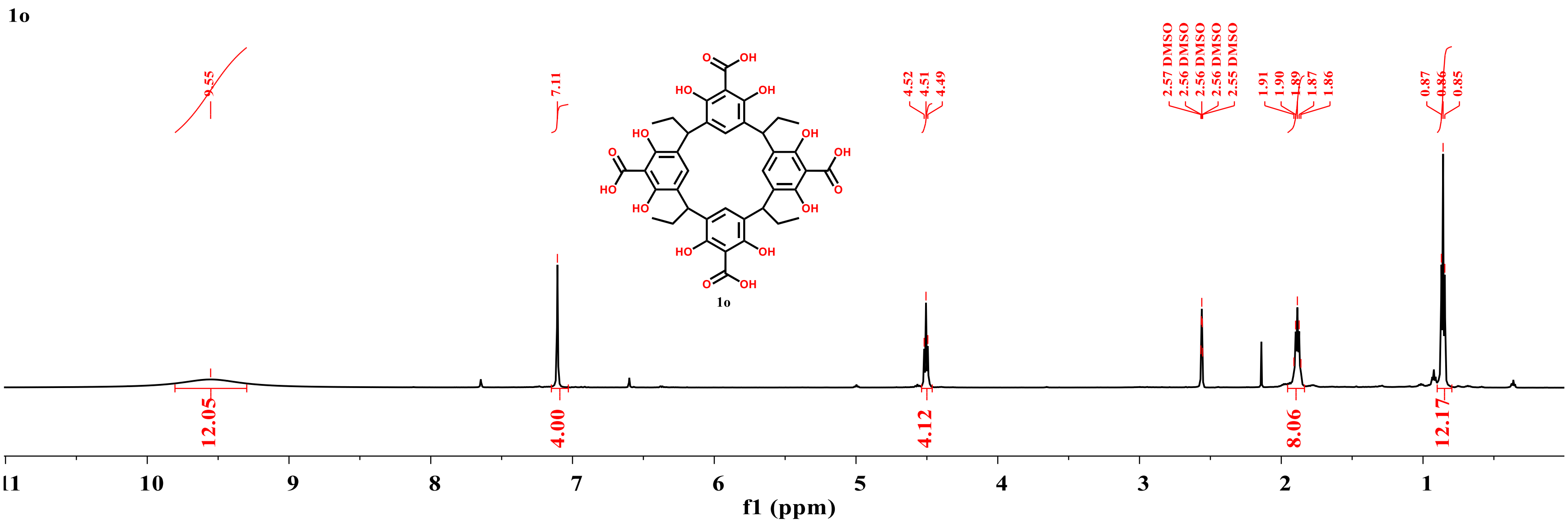

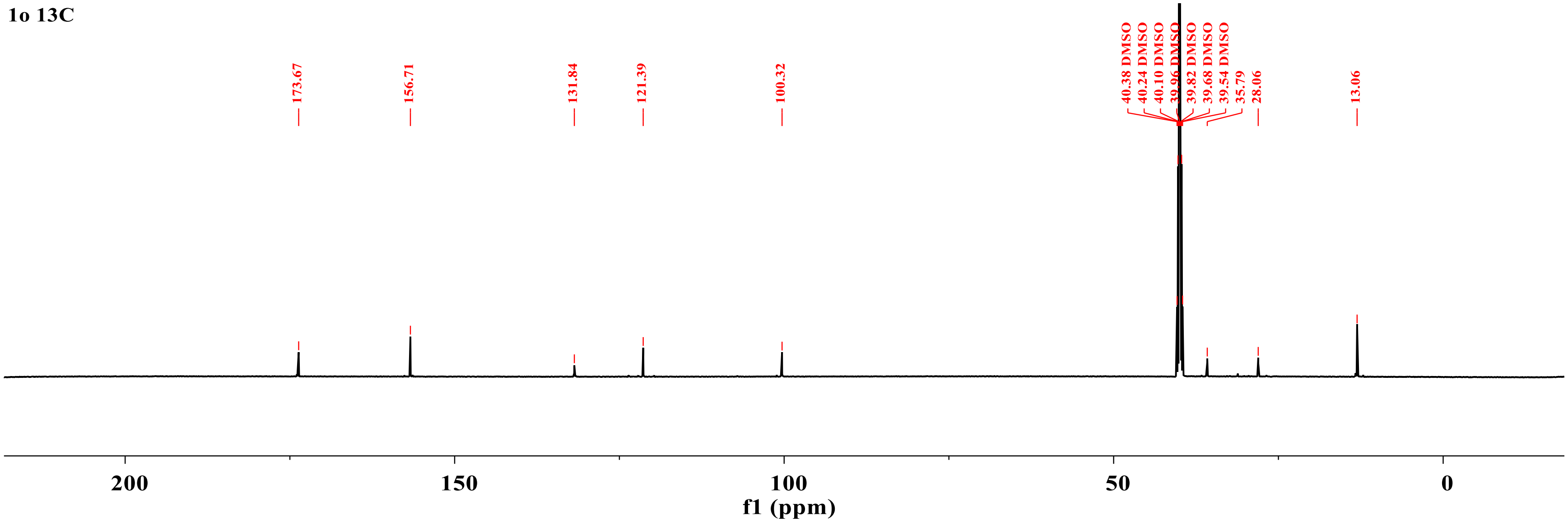

Figure S16. ^{1}H and ^{13}C NMR spectra of compound **1o**. Data collected in DMSO-d_6 at 20 °C.

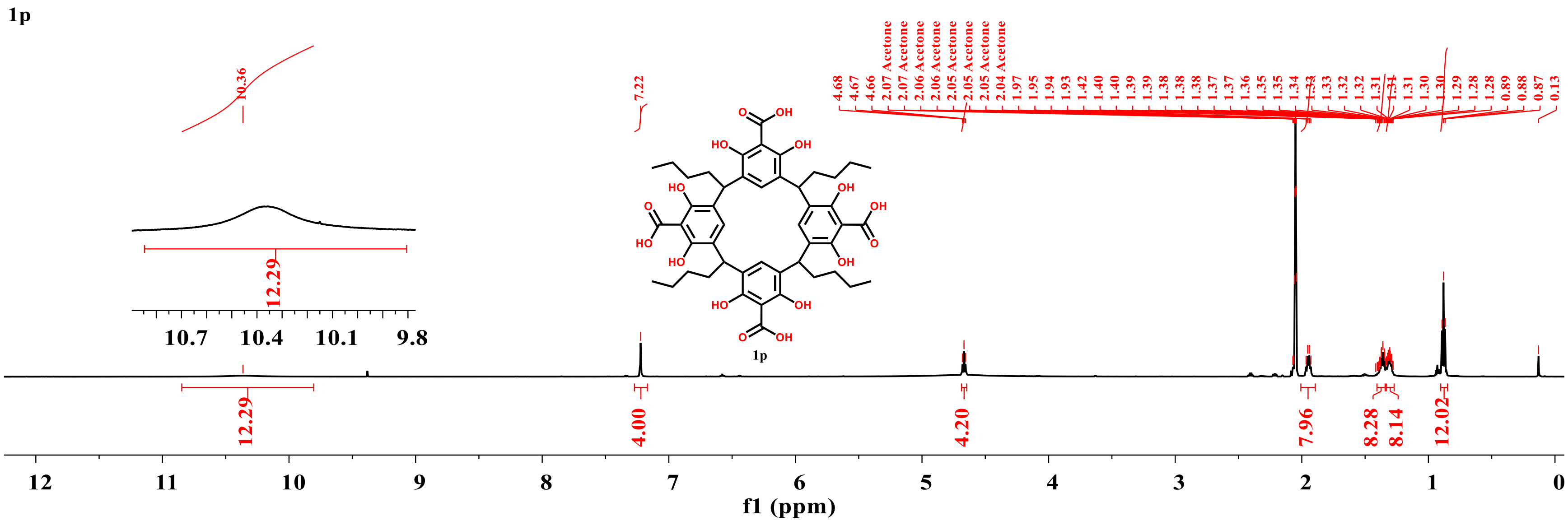

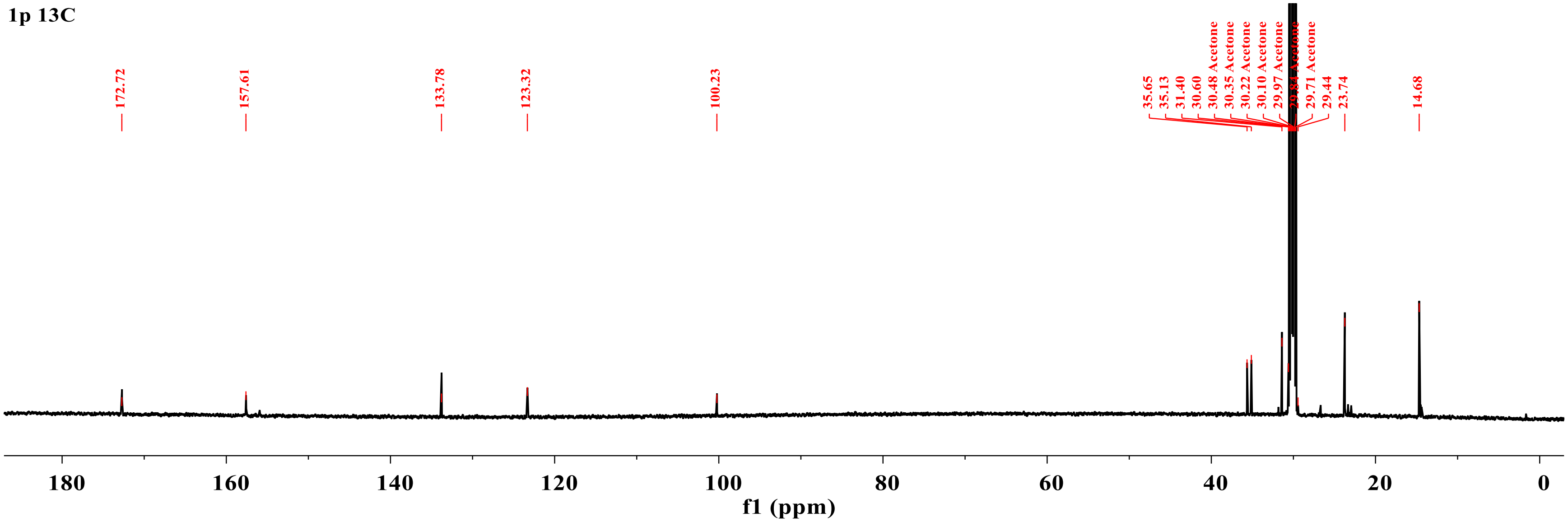

Figure S17. ^{1}H and ^{13}C NMR spectra of compound **1p**. Data collected in acetone-d_6 at 20 °C.

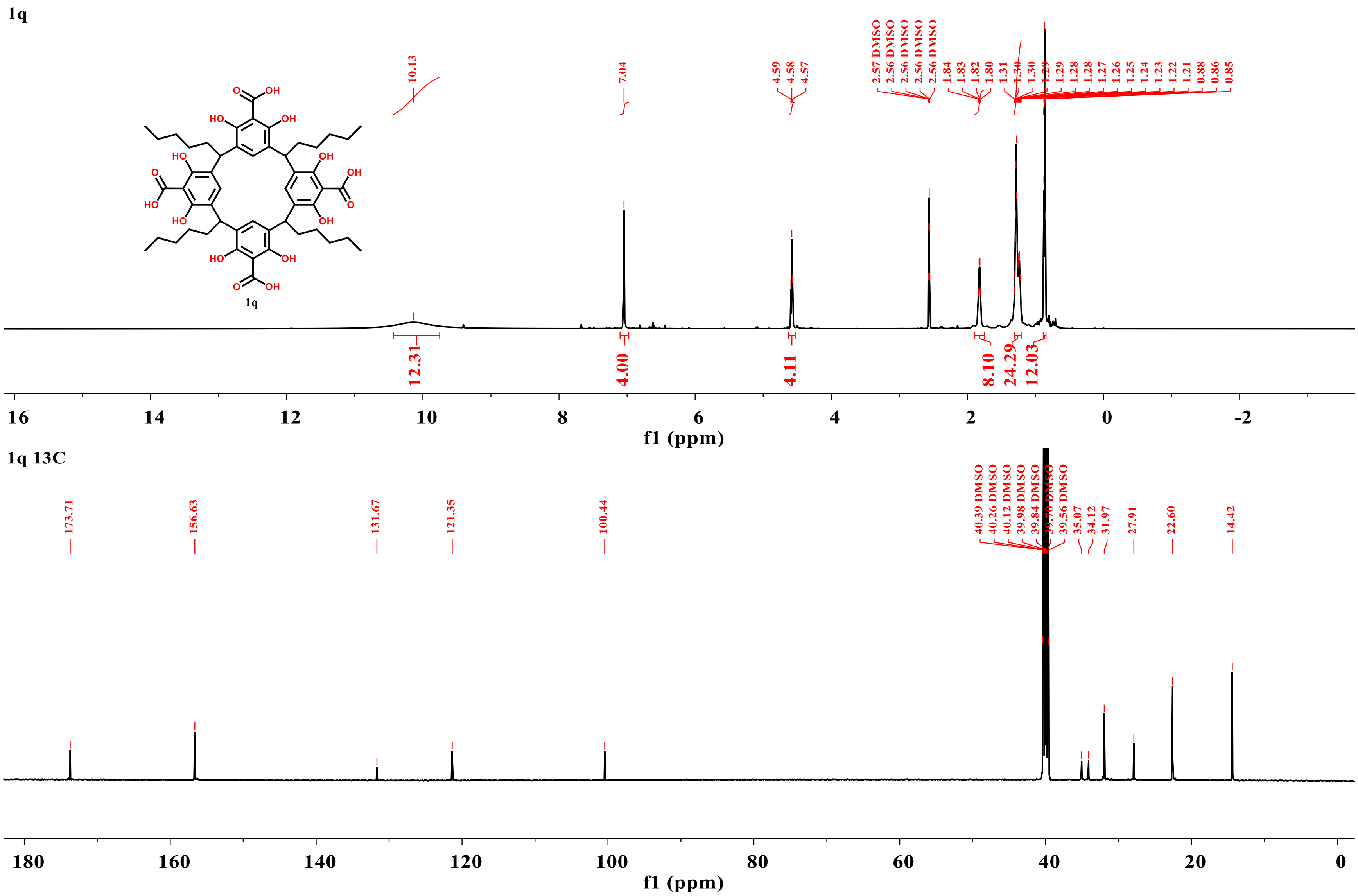

Figure S18. ^{1}H and ^{13}C NMR spectra of compound **1q**. Data collected in DMSO-d_6 at 20 °C.

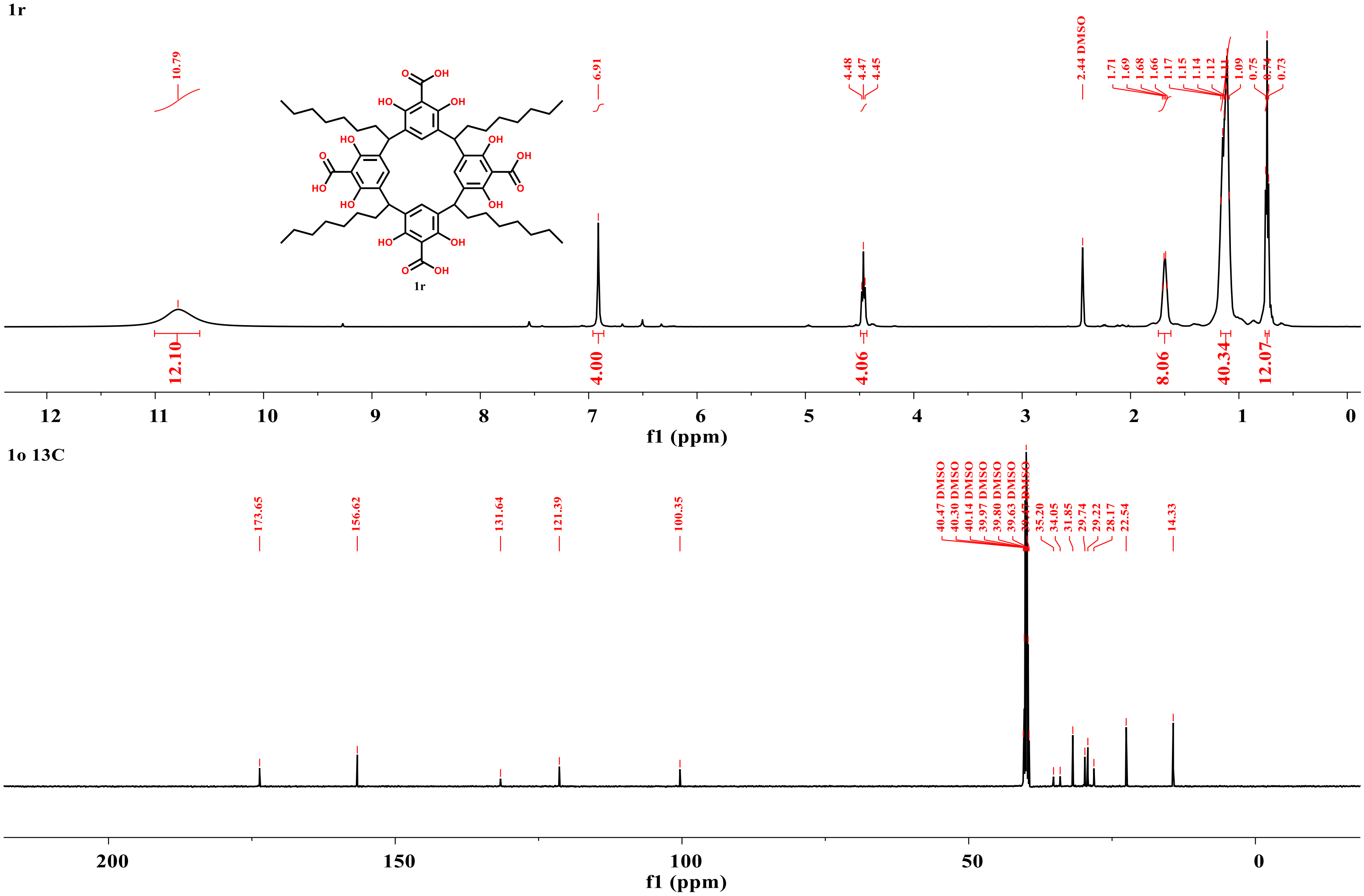

Figure S19. ^{1}H and ^{13}C NMR spectra of compound **1r**. Data collected in DMSO-d_6 at 20 °C.

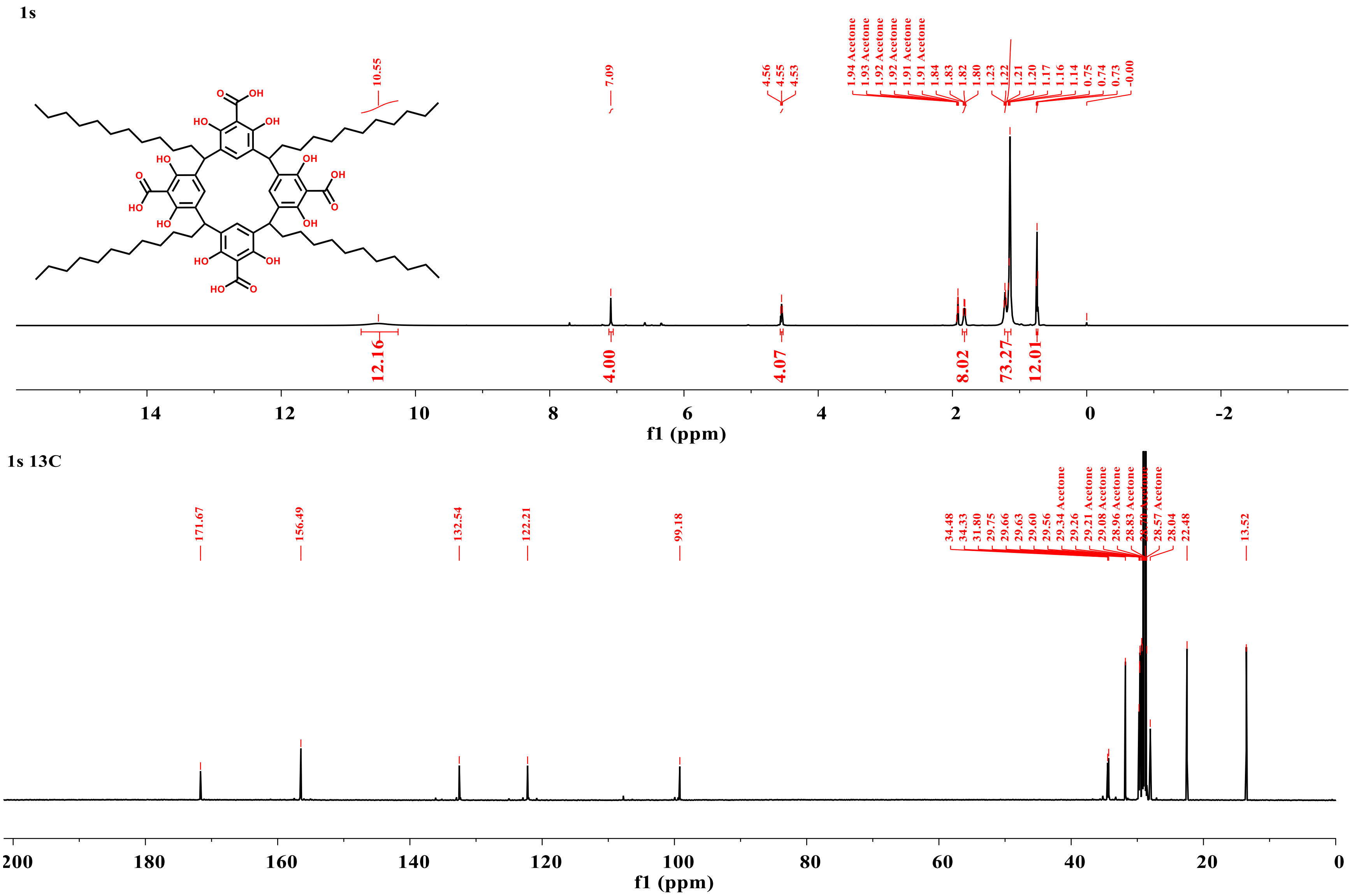

Figure S20. ^{1}H and ^{13}C NMR spectra of compound **1s**. Data collected in acetone-d_6 at 20 °C.

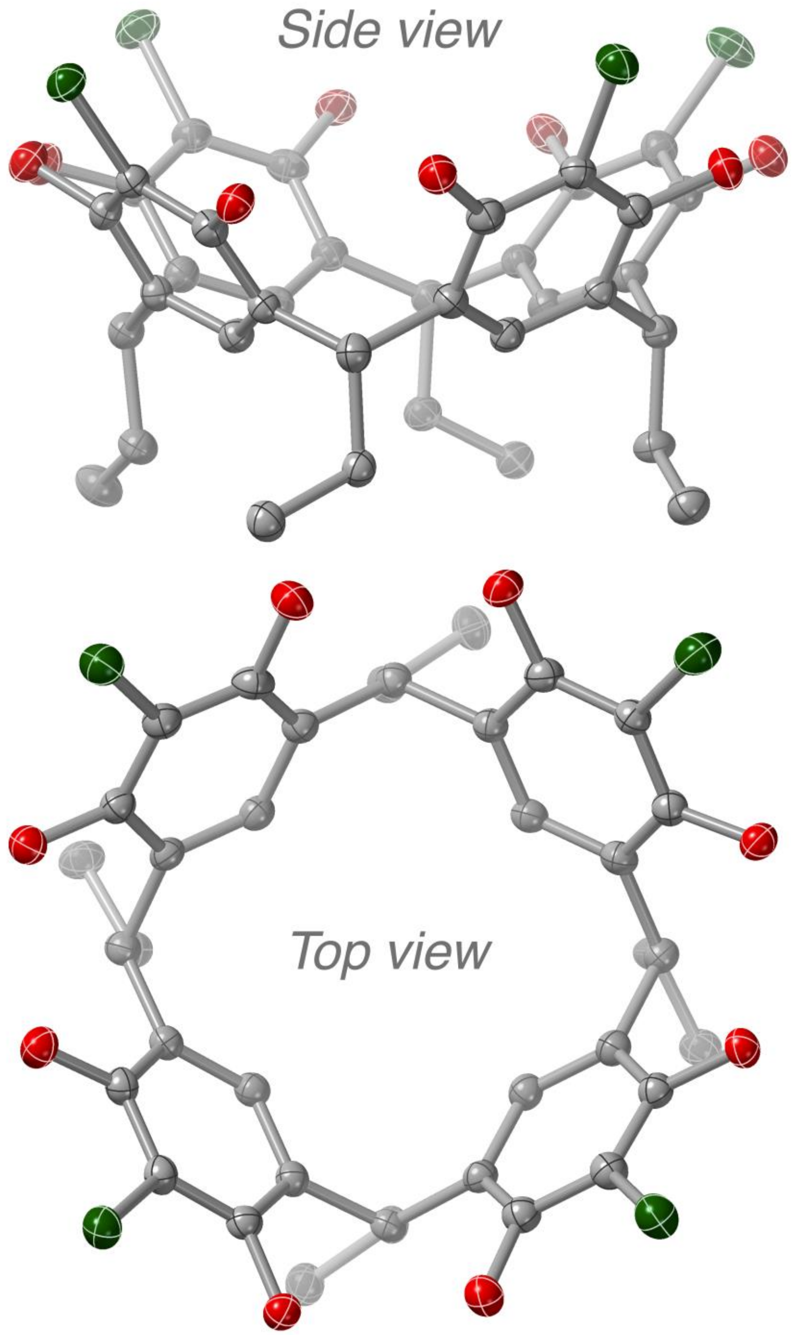

Figure S21. Molecular crystal structure of **1h** obtained at 100 K. Thermal ellipsoids are set at 50% probability level. Hydrogen atoms are omitted for clarity. The C, Cl, and O atoms are colored grey, green, and red, respectively.

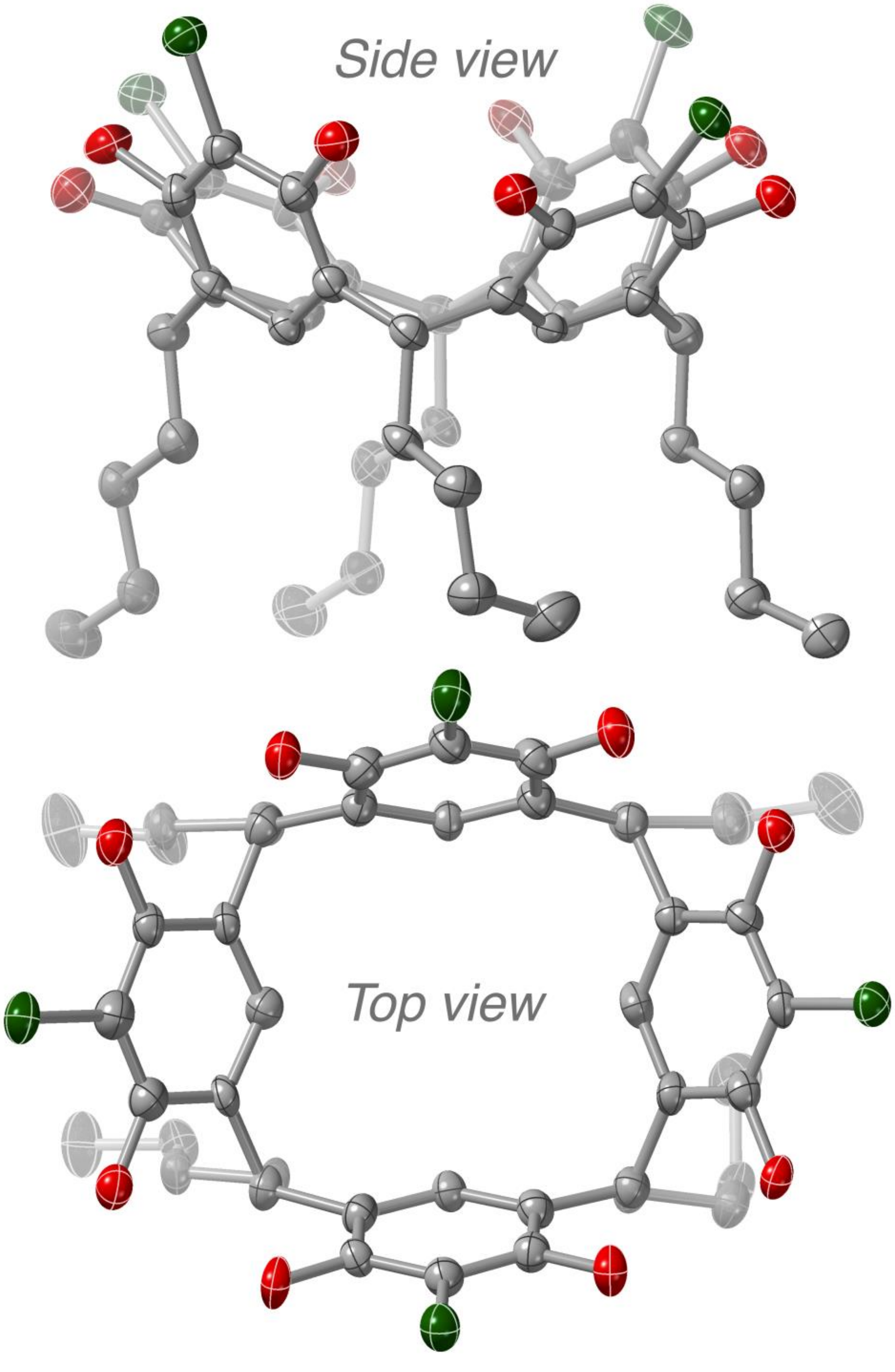

Figure S22. Molecular crystal structure of **1i** obtained at 100 K. Thermal ellipsoids are set at 50% probability level. Hydrogen atoms are omitted for clarity. The C, Cl, and O atoms are colored grey, green, and red, respectively.

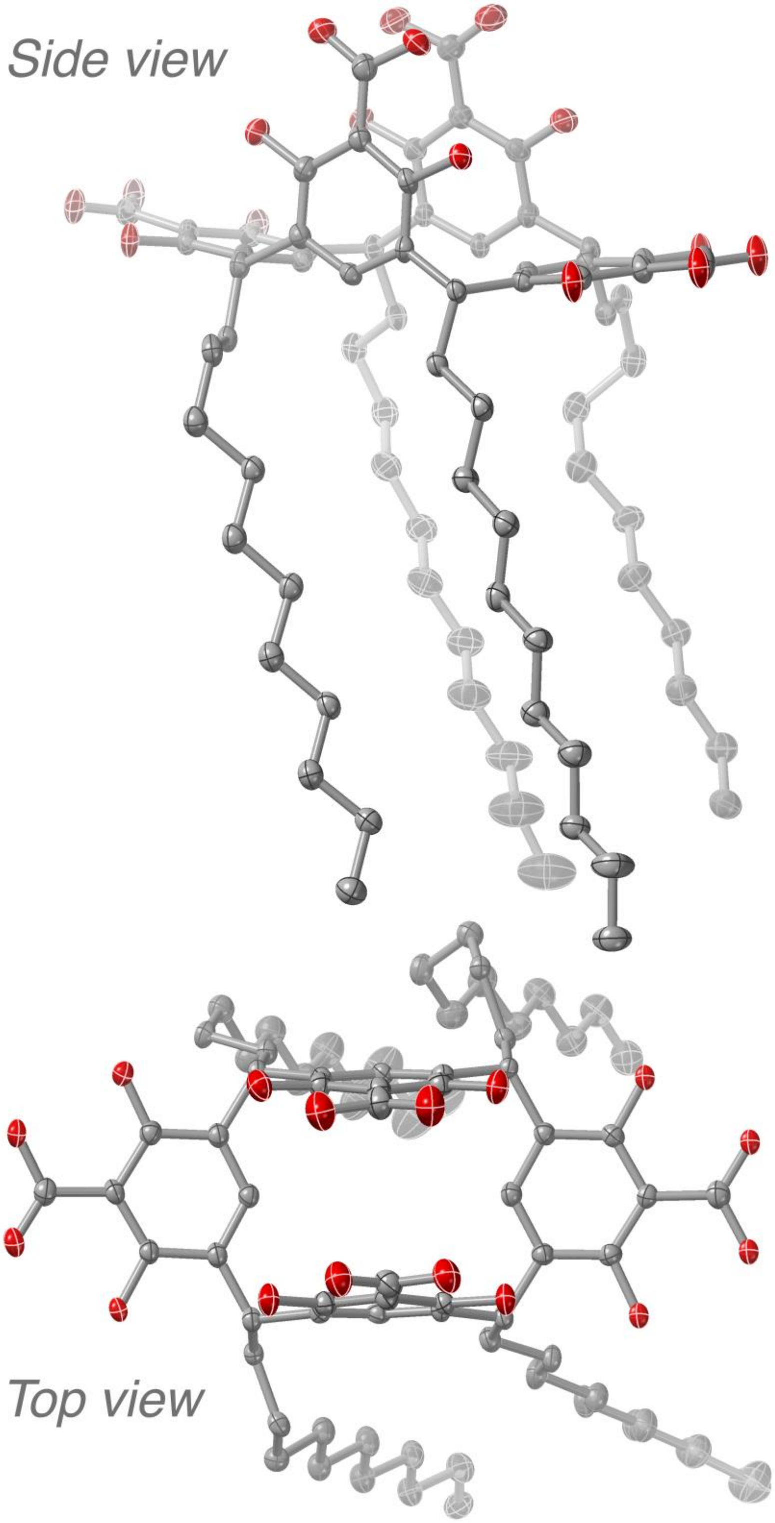

Figure S23. Molecular crystal structure of **1s** obtained at 100 K. Thermal ellipsoids are set at 50% probability level. Hydrogen atoms are omitted for clarity. The C and O atoms are colored grey and red, respectively.

References

1. Spek, A. *J. Appl. Cryst.* **2003**, *36*, 7-13.
2. Spek, A. *Acta Cryst. D* **2009**, *65*, 148-155.
3. Sheldrick, G. M. *Acta Cryst. A* **2015**, *71*, 3-8.
4. *Acta Cryst. C* **2015**, *71*, 3-8.
5. Dolomanov, O. V.; Bourhis, L. J.; Gildea, R. J.; Howard, J. A. K.; Puschmann, H. *J. Appl. Cryst.* **2009**, *42*, 339-341.
6. Tunstad, L. M.; Tucker, J. A.; Dalcanale, E.; Weiser, J.; Bryant, J. A.; Sherman, J. C.; Helgeson, R. C.; Knobler, C. B.; Cram, D. J. *J. Org. Chem.* **1989**, *54*, 1305-1312.
7. Salas, S. D.; Baumgartner, M. T.; Veglia, A. V. *J. Mol. Liq.* **2019**, *277*, 769-775.
8. Adhikari, B. B.; Fujii, A.; Schramm, M. P. *Eur. J. Org. Chem.* **2014**, *2014*, 2972-2979.
9. Fransen, J. R.; Dutton, P. J. *Can. J. Chem.* **1995**, *73*, 2217-2223.
10. Hussain, H.; Du, Y.; Tikhonova, E.; Mortensen, J. S.; Ribeiro, O.; Santillan, C.; Das, M.; Ehsan, M.; Loland, C. J.; Guan, L.; Kobilka, B. K.; Byrne, B.; Chae, P. S. *Chem. Eur. J.* **2017**, *23*, 6724-6729.
11. Tan, S.-D.; Chen, W.-H.; Satake, A.; Wang, B.; Xu, Z.-L.; Kobuke, Y. *Org. Biomol. Chem.* **2004**, *2*, 2719-2721.
12. Ananthnag, G. S.; Mondal, D.; Mague, J. T.; Balakrishna, M. S. *Dalton Trans.* **2019**, *48*, 14632-14641.
13. Bryant, J. A.; Blanda, M. T.; Vincenti, M.; Cram, D. J. *J. Am. Chem. Soc.* **1991**, *113*, 2167-2172.
